# Potential Role of Decoy B7-H4 in the Pathogenesis of Rheumatoid Arthritis: A Mouse Model Informed by Clinical Data

**DOI:** 10.1371/journal.pmed.1000166

**Published:** 2009-10-20

**Authors:** Takeshi Azuma, Gefeng Zhu, Haiying Xu, A. Cecilia Rietz, Charles G. Drake, Eric L. Matteson, Lieping Chen

**Affiliations:** 1 Department of Oncology, Johns Hopkins University School of Medicine, Baltimore, Maryland, United States of America; 2 Division of Rheumatology, Mayo Clinic, Rochester, Minnesota, United States of America; The University of Texas M.D. Anderson Cancer Center, United States of America

## Abstract

**Background:**

A pathogenic hallmark of rheumatoid arthritis (RA) is persistent inflammatory responses in target tissues and organs. Immune responses mediated by T cells and autoantibodies are known to play pivotal roles. A possible interpretation for this observation is a loss of negative regulation of autoimmune responses. Here we sought to investigate whether B7-H4, a cell surface inhibitory molecule of the B7-CD28 signaling pathway, may play a role in the pathogenesis of RA.

**Methods and Findings:**

In a cross-sectional study of a clinical convenience sample using monoclonal antibodies against human B7-H4 molecules, we detected high levels of the soluble form of B7-H4 (sH4) in the sera of 65% of patients with RA (*n* = 68) versus only 13% of healthy donors (*n* = 24). Elevated sH4 was associated with an increased disease severity score (DAS28) in a cross-sectional analysis. In a mouse model of RA, transgenic expression of sH4 or genetic deletion of B7-H4 accelerated the progression of collagen-induced arthritis, accompanied by enhanced T and B cell–mediated autoimmune responses as well as increased activity of neutrophils. Expression in vivo of an agonist, a B7-H4-immunoglobulin Fc fusion protein, profoundly suppressed disease progression in the mouse model.

**Conclusions:**

Our findings in mice indicate that sH4 acts as a decoy molecule to block the inhibitory functions of cell-surface B7-H4, leading to exacerbation of collagen-induced arthritis. If the preliminary correlation between sH4 levels and disease activity in patients with RA can be confirmed to reflect a similar mechanism, these findings suggest a novel target for treatment approaches.

*Please see later in the article for the Editors' Summary*

## Abstract 2

Finding an association between soluble B7-H4 and rheumatoid arthritis severity, Lieping Chen and colleagues use a mouse model to show that the soluble form blocks the inhibitory function of cell-surface B7-H4.

## Editors' Summary

**Background:**

Rheumatoid arthritis (RA) is a chronic disease caused by abnormal immune responses. In RA, the body's own immune system mainly attacks the joints, causing inflammation in their lining, but can affect other tissues and organs in the body. About 1% of the population in developed countries suffer from RA, and it can result in long-term joint damage, causing significant illness and disability. Sufferers have chronic pain, loss of function of the joint, and loss of mobility. The cause of RA is unknown and there is no known cure. However, neutrophils (an immune cell important for inflammation) are thought to contribute to the initiation of RA. Understanding the primary mechanisms behind the development of RA, and where the body's immune system goes wrong, is fundamental not only to find new treatments for the disease but also to aid diagnosis to help patients get treatment to help control their often debilitating symptoms.

**Why Was the Study Done?:**

Regulation of the immune system is necessary to prevent overactivity. Interruptions to the normal signals that moderate the immune response can lead to destruction of normal tissues. Previous studies have shown that the B7 family of proteins, which interact with CD28 signaling proteins on the surface of immune cells, are important regulators of the immune response. B7 proteins have also been found to exist in soluble forms that have been implicated in the development of rheumatoid diseases, but their exact role is not well understood. In the current study, researchers examined a member of the B7 family, B7-H4, which normally acts as an inhibitor of the immune response, to find out whether this signaling molecule affects the immune response and has a role in the development of RA.

**What Did the Researchers Do and Find?:**

The researchers collected blood from 68 patients with RA and 24 healthy volunteers, and measured levels of soluble B7-H4, also known as sH4. They found sH4 in blood from 65% of patients with RA, compared with only 13% of healthy people. The levels of sH4 were significantly higher in RA patients (96.1 ng/ml) compared to healthy people (<5 ng/ml). Moreover, the highest levels of sH4 were found in patients with the most severe forms of RA, as measured by a standard index score that includes general health, the number of swollen joints, and the amount of inflammation. The researchers then used a mouse model of RA to explore how sH4 might contribute to RA. First, they injected mice with plasmids (circular pieces of DNA that can be used to transfer genes into organisms) carrying the gene for sH4 and looked at how overexpression of sH4 affected the development of arthritis. They also looked at how deleting the B7-H4 gene in mice affected symptoms. Both overexpression of sH4 and deletion of B7-H4 caused inflammation in the mice; symptoms appeared earlier and were more severe. Furthermore, the effects of sH4 were shown to be dependent on neutrophils. Finally, the researchers successfully prevented the development of disease in mice by using a protein to mimic the normal signaling by B7-H4, which inhibits the immune response.

**What Do these Findings Mean?:**

These findings suggest that the signaling molecule B7-H4 may be involved in the development of RA. B7-H4 normally acts as an inhibitor of the immune response to suppress inflammation, but when its action is blocked the immune response is no longer suppressed, and an inappropriate and increased immune reaction occurs. sH4 is thought to act as a decoy that blocks binding of B7-H4 to its receptor, thereby preventing an inhibitory signal to the immune system. Overexpression of sH4 worsens the symptoms in the mouse model of RA. Intriguingly, high levels of sH4 were also present in RA patients and were associated with increased severity of disease. This study does not establish sH4 as a cause of RA but implicates sH4 as a cause in the progression of increased inflammation in this disease. Immune system signaling molecules have potential as novel targets for treatment of RA and other autoimmune disorders. However, further studies are needed to test whether sH4 has a direct role in the development of RA in humans.

**Additional Information:**

Please access these Web sites via the online version of this summary at http://dx.doi.org/10.1371/journal.pmed.1000166.

- MedlinePlus has a topic page on RA providing extensive information on symptoms and treatment for RA and access to related clinical trials and medical literature
- The National Rheumatoid Arthritis Society (UK) is a patient-led charity to provide information, education, and support for people with RA
- The Arthritis Foundation (US) is a national not-for-profit organization that supports public health education and research funding, and provides informational resources for people with arthritis http://www.arthritis.org/

## Introduction

Chronic and persistent inflammation is a major cause for the pathogenesis and progression of systemic autoimmune diseases such as rheumatoid arthritis (RA). During progression of RA, the synovial lining layer of the inflamed joints increases its thickness as a result of synovial hyperplasia and inflammation [1],[2]. The major model for RA is collagen-induced arthritis (CIA) in which challenge of DBA/1j mice with type II chicken collagen (CII) induces persistent chronic inflammation in all major joints [3]. While CD4+ T cells were considered to play a central role in the pathogenesis of RA, neutrophils may also contribute to initiation, progression, and maintenance of RA [4]–[6]. For example, synovial fluid from patients with active RA is heavily infiltrated with neutrophils [7]. Elevated levels of granulocyte colony stimulating factor (G-CSF), a major growth factor for neutrophils, were detected in sera and synovial fluid from RA patients and correlated with disease activity and severity [8]. In arthritis animal models, depletion of Gr-1+ neutrophils resulted in a decrease of arthritis severity [9]–[14]. In addition, G-CSF is also responsible for trafficking of neutrophils into inflamed joints during progression of CIA [15]. In experimental models, massive infiltration of Gr-1+ cells in the lesions, especially in the early phase, releases proinflammatory cytokines including tumor necrosis factor-alpha (TNF-α), interleukin-1 (IL-1), and IL-6, which can affect the functions of neutrophils and other inflammatory cells [16],[17]. On the other hand, neutrophils could simply play a role as effector cells to increase inflammation. In contrast with the CIA model, however, neutrophil infiltration is less evident in late stages of human RA [7].

Co-signaling molecules, including those with costimulatory and coinhibitory functions, are important for the induction of an effective immune response and for the prevention of unwanted autoimmunity [18]. It has been shown that signals through the B7-CD28 pathway are major regulators of this balance and play a pivotal role in the regulation of autoimmunity [19],[20]. Persistence of inflammatory responses in systemic autoimmune diseases implies either impaired coinhibitory or enhanced costimulatory functions, leading to the loss of balance. In this regard, it is particularly interesting that autoantibodies against B7-H1, a primary coinhibitory molecule, are found in a significant proportion of patients with RA and implicated in the progression of RA symptoms [21].

Soluble forms of B7-CD28 family molecules are also implicated in the progression of rheumatoid diseases. A recent study shows that soluble programmed death one (PD-1) could be detected in RA patients and the levels of soluble PD-1 are correlated with TNF-α concentration in synovial fluid [22]. The role of these soluble molecules in disease progression, however, remains to be established. B7 homolog 4 (B7-H4) is a more recently identified member of the B7 family with potent inhibitory effects in T cells through binding to a putative receptor [23],[24]. Cell surface B7-H4 is normally not detectable in normal tissues, but its surface expression could be up-regulated on macrophages and tumor cells by inflammatory cytokines including IL-10 and IL-6 [25]. Our group and others reported that B7-H4 could suppress T cell response in the presence of antigen stimulation [23],[26],[27]. Soluble B7-H4 (sH4) was also detected in ovarian cancer patients as a potential biomarker, but the mechanism of production and the function of sH4 are unknown [28]. B7-H4-deficient mice have been found to mount slightly enhanced T helper 1 type T cell responses against *Leishmania major* infection [29]. Our study using independently generated B7-H4-knockout (B7-H4KO) mice demonstrated that the lack of B7-H4 led to resistance to *Listeria monocytogenes* infection by lifting suppression on the growth of neutrophil progenitors [30]. Taken together, these studies indicate that B7-H4 is an important inhibitory molecule to keep the inflammatory response in check.

In this study we investigated whether sH4 could be detected in a higher quantity in the sera from RA patients than healthy donors (HDs), and whether levels were associated with disease activity. The results lead us to postulate that sH4 acts as a decoy to impair endogenous B7-H4-mediated suppression of inflammatory responses. We then tested this hypothesis in a CIA mouse model.

## Materials and Methods

### Patients and HDs

Patients were recruited from a convenience sample enrolled serially as they were seen in the outpatient clinic. Diagnoses of RA [31] are based on the Criteria for Classification of Rheumatoid Arthritis by the American Rheumatism Association. The HDs had no history of autoimmune diseases and were recruited similarly. The study and protocol were approved by the Internal Review Board of the Mayo Clinic and all patients and HDs gave written informed consent for this study. The characteristics of RA patients, including anti-nuclear antibody (ANA), absolute neutrophil count (ANC), C-reactive protein (CRP), and clinical treatment, are summarized in Table S1. The disease activity score 28 (DAS28) ranges from 0 to 10 and includes the 28 tender and swollen joint counts, the erythrocyte sedimentation rate (Westergren, mm/h), and the patient's general health measured with a visual analog scale (100 mm) [32]. DAS28>5.1 indicates that the patient has high disease activity, DAS28 of 3.3 to 5.1 means that disease activity is moderate, DAS of 2.7 to 3.2 is categorized as low disease activity, and DAS<2.6 indicates remission. The DAS assessment was done shortly prior to the blood draw (within 2 h). The drugs for RA treatment were taken at the time of the DAS evaluation and blood draw.

### Detection of sH4 and Autoantibodies against Collagen

For detection of human sH4, specific monoclonal antibodies (mAbs) hH4.3 (2 µg/ml) and hH4.1 (2 µg/ml) against human B7-H4 [24] were used as capture and detection, respectively, by sandwich ELISA. To remove rheumatoid factor, the sera were treated with human IgG agarose (Sigma-Aldrich, St. Louis, MO) before detection by ELISA. After this treatment, sera do not react to human/rat IgG, indicating complete elimination of potential cross-reactivity. For measurement of collagen-specific autoantibodies, chicken collagen (1 µg/ml) was coated on the plate overnight at 4 °C, and biotin-conjugated anti-mouse IgG, IgG1, IgG2a, and IgG2b antibodies (BD, San Jose, CA) were used as detection antibodies. ELISA was conducted according to the procedures described previously [21].

For detection of mouse sH4 by sandwich ELISA, specific mAb, clone mH4.5 [23] at 2 µg/ml, was used as capture antibody. As detection antibody, polyclonal antibodies were prepared by immunization of a rat with peptides encoding B7-H4 IgV domain-KLH conjugate, as in the procedure described previously [33]. All sera were pretreated with mouse IgG agarose (Sigma-Aldrich) to remove rheumatoid factor before ELISA.

### Western Blot

The sera were mixed with 2× sample buffer (4% SDS, 0.2% bromophenol blue, 20% glycerol in 100 mM Tris-buffered saline) and boiled for 5 min. The samples were electrophoresed under reducing conditions on a 10% Ready Gel (Bio-Rad, Richmond, CA) and the proteins electroblotted onto Protran BA85 (Whatman, Florham Park, NJ). The Immobilon-P sheet was blocked in 5% nonfat dry milk in PBS for 1 h and incubated with the antibody (clone hH4.1) at 4°C overnight. After repeated washing (five times for 5 min), bound antibody was detected with horseradish peroxidase (HRP)–labeled streptavidin (Biosource, Camarillo, CA), incubated for 1 h, and visualized by chemiluminescent substrate (Supersignal Substrate, Pierce, Rockford, IL).

### Mice

Male DBA/1j mice were purchased from Jackson Laboratory (Bar Harbor, ME). Age-matched mice, 4–10 wk old, were used for all experiments. B7-H4KO mice were generated from 129/B6 embryo stem cells in our laboratory [30] and have been backcrossed to B6 background for 10 generations. DBA/1j×B7-H4KO mice were generated by backcrossing B7-H4KO mice into DBA/1j backgrounds for eight generations. Two markers on the upstream of B7-H4, D3mit21 (19.2 cM), and D3mit278 (33.7 cM) and two markers on the downstream of B7-H4, D3mit318 (58.8 cM), and D3mit127 (70.3 cM) are all DBA/1 products (unpublished data). All mice were maintained in the Animal Facility at Johns Hopkins University under a protocol approved by the Institutional Animal Care and Use Committee.

### Antibodies and Flow Cytometry Analysis

Purified mAbs against mouse Gr-1 (RB6-8C5) and CD11b (M1/70) were purchased from PharMingen (San Diego, CA). Fluorescence was detected by FACScan flow cytometry and analyzed with Cell Quest software (Becton Dickinson, Mountain View, CA).

### Induction of CIA

CIA was induced in 8- to 10-wk-old male DBA/1j mice by intradermal tail base injection of 0.2 mg chicken collagen (Sigma-Aldrich) in 0.05 M acetic acid, supplemented with 4.0 mg/ml *Mycobacterium tuberculosis* (DIFCO, Detroit, MI) emulsified in complete Freund adjuvant. Twenty-one days after first primary immunization, the mice were identically boosted once. Severity of disease was evaluated by visual inspection of the paws. Each paw was scored for the degree of inflammation on a scale from 0 to 4 where 0, no evidence of erythema and swelling; 1, erythema and mild swelling confined to the midfoot (tarsals) or ankle joint; 2, erythema and mild swelling extending from ankle to the midfoot; 3, erythema and mild swelling extending from ankle to metatarsal joints; and 4, erythema and severe swelling encompassing the ankle, foot, and digits. Scores from all four paws were added to give the total for each animal.

### Murine B7-H4 Constructs

The B7-H4 immunoglobulin fusion (B7-H4Ig) construct was described previously [23]. To generate plasmids of B7-H4V, which contains only Ig V region of B7-H4, and B7-H4VC, which have both Ig V and Ig C regions, , two flanking 5′ and 3′ primers were designed with XhoI and EcoRI restriction sites, respectively (5′ primer, 5′-CCGCTCGAGCCACCATGGCTTCCTTGGGGCAG-3′; 3′ primer for B7-H4V, 5′-CGGAATTCCGCTAATTTATCTCTGGCATACT-3′; 3′ primer for B7-H4VC, 5′-CGGAATTCCGCTAAGAGTTCAGCAACTGCAG-3′). Appropriate regions of cDNA were amplified using primers. PCR product was digested with XhoI and EcoRI and ligated into XhoI/EcoRI-digested pcDNA3.1 vectors (Invitrogen, Carlsbad, CA).

### Hydrodynamic Injection and Antibody Treatment

Mice were injected with indicated plasmid on day −1 and day 13 in the CIA model. Hydrodynamic injection was performed as previously described [34]. Briefly, 30 µg of plasmid DNA in 3 ml of PBS was injected into the tail vein within 10 s. Mouse survival was 100% after injection. To delete granulocytes, mice were injected i.p. with 0.3 mg of anti-Gr-1 mAb (clone RB6-8C5, rat IgG2b). The controls that were used in the study were either control polyclonal rat IgG (Sigma) or anti-human HLA-Bw6 mAb (clone SFR8-B6, rat IgG2b) every other day.

### Collagen-Specific T Cell Proliferation and Cytokine Production

The spleen was removed on day 14 after the last immunization. CD4+ T cells were purified by using magnetic beads (Miltenyi Biotec, Auburn, CA). Whole splenocytes or purified CD4+ T cells were stimulated with denatured (60°C, 30 min) CII in 96 well flat bottom microtiter plates for 72 h, and pulsed with [^3^H] thymidine (1 µCi/well) (Amersham Pharmacia Biotech, Piscataway, NJ) for the final 12 h. In the culture of purified CD4+ T cells, irradiated (50 Gy) splenocytes from the syngeneic mice were added as antigen-presenting cells. Supernatants from the cultures were collected after 48 h and assayed for mouse IFN-γ (BD) and IL-17A (eBioscience) using the ELISA kit according to the protocols recommended by the manufacturer.

### Histological Assessments of Arthritis

CIA mice were humanely killed at day 35. The hind paws were removed, fixed in formalin, decalcified in 10% EDTA, embedded in paraffin, sectioned, and stained with hematoxylin and eosin.

### Statistical Analysis

We performed statistical analysis with the Mann-Whitney U test for single comparison and ANOVA followed by the Scheffé test for multiple comparisons. Correlations were performed with the Spearman rank test. For longitudinal multiple comparisons, repeated ANOVA followed by the Tukey-Kramer test was used. The unpaired Student two-tailed *t*-test was applied for single comparison of parametric data. Independent associations between one dependent variable and more than two independent variables were assessed by multiple regression analysis. Specifically, the dependent variable, sH4 concentration, was regressed for RA, age, and sex. In all statistical analyses, significance was accepted at *p*<0.05.

## Results

### Increased sH4 in the Sera from Patients with RA

The characteristics of RA patients, including their age, sex, disease duration, ANA, ANC, CRP, sH4 levels, clinical treatment, and DAS28 score, are summarized in Table S1. Serum samples were obtained from 68 patients with diagnosed RA (55 women and 13 men, mean age = 56 y; age range: 19–80 y) and 24 HDs (19 women and 5 men, mean age = 54 y; age range: 20–69 y) as controls. The treatment history of patients before and after admission was listed in Table S1.

**Figure 1:**
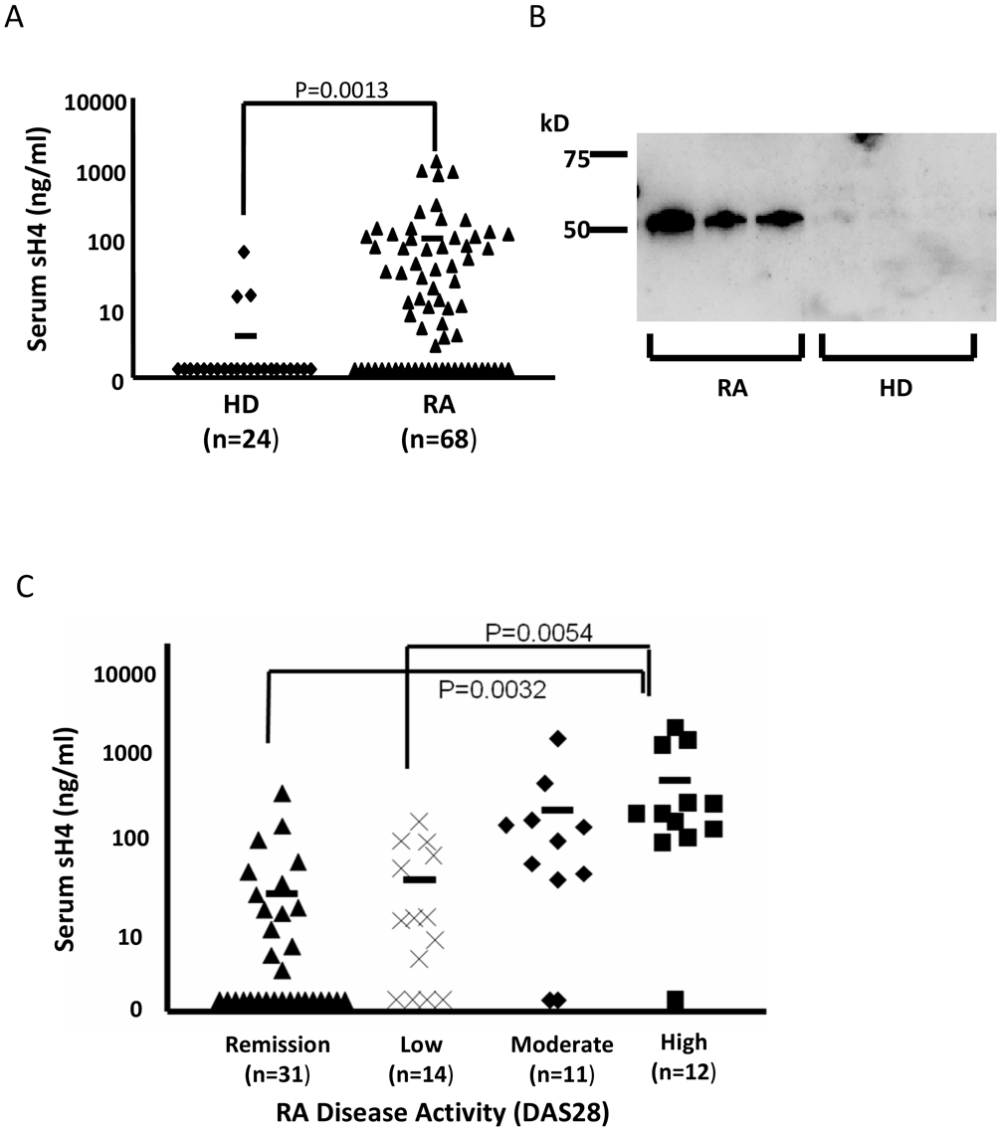
Detection of sH4 in sera from RA patients. (A) The concentration of the sH4 in HDs and in the patients diagnosed with RA. Sera from 68 RA patients and 24 HDs were diluted 1∶10 in PBS and tested by ELISA as described in Materials and Methods. The data were analyzed by the Mann-Whitney U test followed by a multiple regression analysis (*p*<0.01). (B) The sH4 in sera of RA patients was detected by Western blotting using HRP-labeled anti-human B7-H4 mAb (clone hH4.3). A 50 kDa sH4 band was detected in the sera of three patients with RA but not in the sera from three HDs. (C) The correlation of serum sH4 levels and disease severity in the patients with RA. Patients with RA were classified into four severity groups based on DAS28 score described in the Materials and Methods. The concentration of sH4 in the serum of each patient was determined by ELISA as described above. Statistical analysis was performed by ANOVA method followed by the Scheffé test. The bars represent the mean concentration of sH4 in all donors of each group (ng/ml).

To detect sH4, sera from individual patients with a diagnosis of RA were analyzed by ELISA using two specific mAb binding to different epitopes on human B7-H4. In this assay, 65% (44 out of 68) samples from patients with RA were above background for sH4 and therefore considered positive. Evaluation of sH4 in HDs showed only 13% (3 out of 24) to be positive (*p*<0.001) (Figure 1A). The levels of sH4 were also significantly higher in RA patients than HDs: the mean concentration of sH4 in RA (96.1 ng/ml) was significantly higher than those of the HDs (<5 ng/ml) (Figure 1A).

We subsequently performed Western blot analysis to validate the presence of sH4 in sera from three patients with RA. Using specific mAb against B7-H4, the sera from three patients (numbers 27, 38, and 59, see Table S1), who had the highest level of sH4 on ELISA, showed a single 50 kDa band. This matches the size of the predicted extracellular domain of human B7-H4 [23]. In contrast, no band was observed in sera from three HDs, who also had the highest level of sH4 on ELISA (Figure 1A and 1B). Our data support the presence of sH4 in the sera of patients with RA.

### Increased Serum sH4 Is Associated with Active Disease Status

We next determined whether elevated sH4 is associated with the activity of RA. Based on disease activity categorized by the DAS28 score system, 68 patients with RA were classified into four groups with remission patients in the lowest disease activity and with low, moderate, and high activity as described in Materials and Methods. The mean concentration of sH4 in the high activity group (321.6 ng/ml) was significantly higher than those of the remission group (18.93 ng/ml) or low activity group 1 (27.17 ng/ml). However, there was no significant difference among remission, low, and moderate activity group by Scheffé test (Figure 1C). In addition, the presence of sH4 in these patients is also significantly associated with elevated CRP levels in the sera, a nonspecific surrogate marker for ongoing inflammation (Figure S1).

### sH4 Exacerbates CIA in a Mouse Model

To recapitulate and explore the possible role of sH4 in the pathogenesis of RA, we employed a mouse model of CIA. CIA is a well-characterized mouse model for human arthritis, in which injection of CII into male DBA/1j mice induces swelling and progressive inflammation in large and small diarthrodial joints and leads to arthritis. We first tested whether sH4 is present in this model. To do so, we established a specific ELISA using two mAbs against distinct epitopes of murine B7-H4 (see Materials and Methods). The sera from the mice after CII immunization were drawn from the tail vein, absorbed by mouse IgG–conjugated agarose to remove potential rheumatoid factor, and subjected to ELISA analysis. A significant increase of sH4 could be detected up to 300 ng/ml 1 d after immunization in the sera of all mice. This expression, however, was transient and became non-detectable at Day 5 (Figure S2) and did not reappear throughout the entire course of disease progression. Immunization of mice with CFA alone or injection of Con A or LPS, however, did not induce this transient elevation of sH4 in blood (unpublished data), suggesting that sH4 is not a general marker for inflammation. Therefore, we could not establish a correlation between endogenous sH4 and CIA progression in this model.

**Figure 2:**
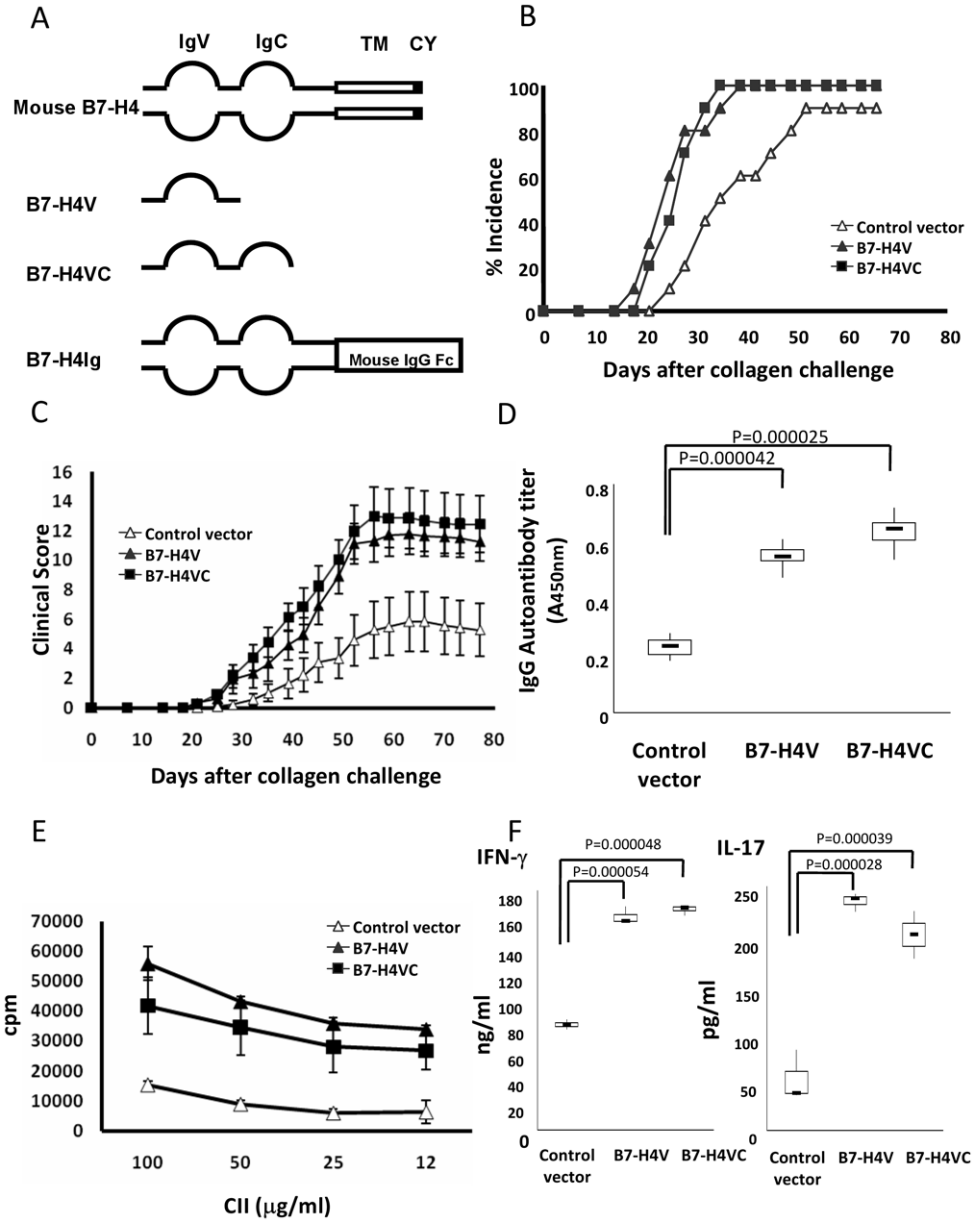
sH4 exacerbates disease in CIA model. (A) Schematic representation of the full length murine B7-H4, B7-H4V, B7-H4VC, and B7-H4Ig fragments, which were inserted into pcDNA plasmid. IgV, immunoglobulin V-like region; IgC, immunoglobulin C-like region; TM, transmembrane region; CT, cytoplasmic tail. Effect of decoy B7-H4 in incidence (B) and (C) clinical score of CIA. Mice were immunized with CII in complete Freund's adjuvant (CFA) on day 0 and day 21. The incidence and clinical score were recorded daily based on the standard described in Materials and Methods. Three groups of mice were injected with the indicated plasmids on day −1 and day 20. Each point represents results from pool of ten mice and expressed as means ± 95% confidence interval. Data represent three independent experiments. Statistical analysis was performed by repeated ANOVA method (*p*<0.0001). The following Tukey-Kramer test showed significant differences at each points after day 28 between the control group and other two groups (*p*<0.01). (D) Effect of decoy B7-H4 in the generation of autoantibodies against CII in CIA sera. CIA mice were treated with indicated plasmids and sera were sampled at day 30 after collagen immunization. Serum levels of IgG against CII were determined by specific ELISA as described Materials and Methods. Each group represents results from a pool of five mice. The top and bottom of each rectangular box denotes the 75th and 25th percentiles, respectively, with the median shown inside the box. Error bars extending from each box represent the maximum and minimum. Data represent three independent experiments. Statistical analysis was performed by ANOVA method followed by the Scheffé test. (E) Effect of decoy B7-H4 in the proliferation of T cells reactive to collagen in CIA mice. CIA mice were first treated with indicated plasmids in vivo as indicated in (B). Whole splenocytes from the mice at day 30 after collagen immunization were cultured in the presence of the indicated concentrations of CII for 72 h. The cultures were pulsed with ^3^HTdR 18 h before harvest, and cpm were determined by a scintillation counter. Each point represents results from three wells and data represent two independent experiments and expressed as means±95% confidence interval. (F) Effect of decoy B7-H4 in the production of cytokines from splenocytes reactive to CII in CIA mice. Supernatants in splenocyte cultures as described in (E) at 72 h were collected and assessed for IFN-γ and IL-17 by ELISA. Each group represents results from three wells and data represent three independent experiments. The top and bottom of each rectangular box denote the 75th and 25th percentiles, respectively, with the median shown inside the box. Error bars extending from each box represent the maximum and minimum. Statistical analysis was performed by ANOVA followed by the Scheffé test.

As an alternative approach to determine the role of sH4, we sought to express sH4 in vivo. To do so, we constructed an expression vector, B7-H4VC, in which the truncated gene encoding both IgV and IgC domains was placed under the control of the cytomegalovirus (CMV) immediate early promoter. We also prepared another vector, B7-H4V, containing only IgV domain of B7-H4 (Figure 2A). After expression, these truncated proteins/polypeptides were expected to compete with endogenous B7-H4 on the cell surface for binding its putative receptor. Parental vector or the vector with the gene encoding flag peptide was included as a control. By a hydrodynamic expression procedure [34], injection of these plasmids led to expression of sH4 up to 2 µg/ml in the sera, based on specific capture sandwich ELISA using two anti-mouse B7-H4 mAbs. The levels of sH4 declined rapidly and become non-detectable by day 7 after injection (Figure S3).

Immunization of DBA/1j mice with CII led to appearance of arthritic symptoms at 25 d. After hydrodynamic injection with control vector, and 80% of mice developed disease on day 49, a result similar to CIA mice without plasmid injection (unpublished data), indicating hydrodynamic injection itself does not significantly change the course of CIA progression. Injection of B7-H4VC vector, however, led to earlier development of disease (18 d after) and 100% of mice developed arthritis at around Day 35. Similar results were also seen in the mice injected with B7-H4V (Figure 2B, 2C). Treatment by either B7-H4V or B7-H4VC also significantly increased severity of arthritis as indicated by increased clinical score (Figure 2C), increased swelling of footpad (Figure S4), and increased infiltration of inflammatory cells in joints as shown in histopathology analysis (Figure S5). Additional control vector containing genes encoding flag peptide or B7-H4-flag fusion protein produced similar results (Figure S6). Our results suggest that decoy B7-H4 could exacerbate CIA and facilitate disease progression.

Assessment of cellular and humoral immune responses revealed that increased incidence and severity of arthritis was accompanied with elevated total IgG autoantibodies (Figure 2D) as well as other subtypes, including IgG_1_, IgG_2a_, and IgG_2b_ against CII at day 30 after immunization and B7-H4VC or B7-H4V treatment (Figure S7). Stimulation of total spleen cells (Figure 2E) or purified CD4+ T cells (Figure S8) from mice, which were treated with B7-H4VC or B7-H4V, by CII also induced much higher levels of proliferation in comparison with mice treated with control vector. Importantly, IFN-γ and IL-17, two major cytokines responsible for CIA progression, also increased significantly in the cultures (Figure 2F). Taken together, our data demonstrate that sH4 enhances autoimmune responses against CII and exacerbates autoimmune CIA.

### B7-H4-Deficient Mice Develop Exacerbated CIA

**Figure 3:**
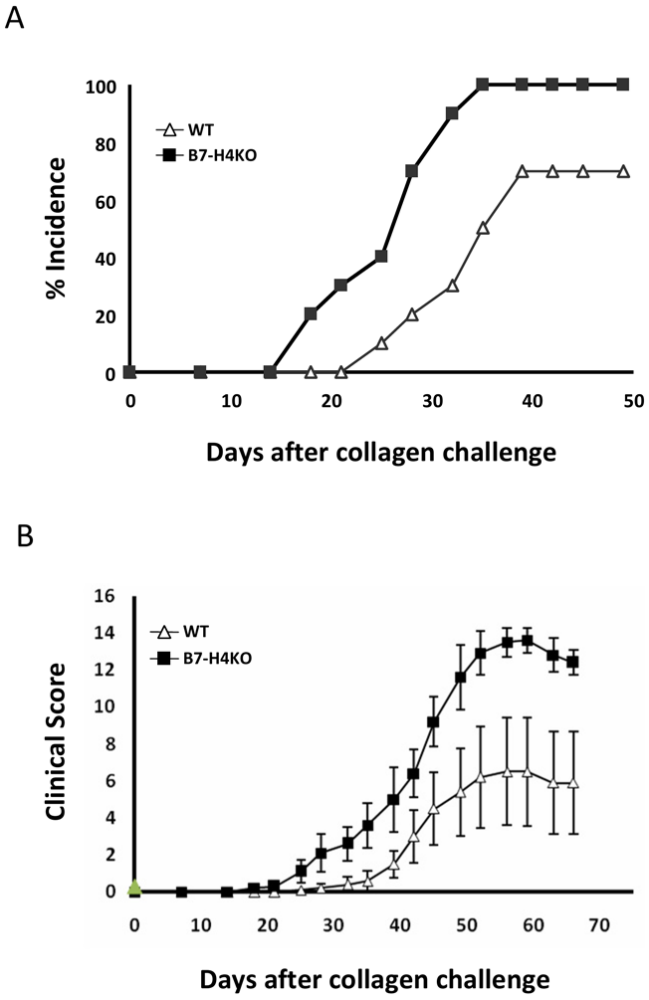
B7-H4-deficient mice develop more severe CIA. (A) CIA incidence and (B) clinical score in the littermate (WT) or B7-H4 knockout (B7-H4KO) mice. The indicated mice were immunized with CII in CFA on day 0 and day 21. Each point represents results from a pool of ten mice. Data represent two independent experiments and expressed as means ± 95% confidence interval. Statistical analysis was performed by repeated ANOVA method (*p*<0.0001). The following Tukey-Kramer test showed significant difference at each points after day 28 (*p*<0.01).

If B7-H4VC and B7-H4V act as decoy molecules to block the effect of endogenous B7-H4 on the cell surface, a prediction is that a similar exacerbating effect should also be observed in the mice lacking endogenous B7-H4. To validate this finding, we backcrossed B7-H4-deficient (KO) mice [30] to DBA/1j mice for eight generations. B7-H4-deficient DBA/1j mice develop normally and do not have obvious abnormalities in gross appearance and immune system development. These mice, however, developed much more severe CIA after CII immunization, showing higher arthritis incidence (Figure 3A) and clinical scores (Figure 3B) than B7-H4+/+ littermates. In addition, autoimmune responses, including T cell responses and autoantibodies to CII, were also significantly increased above control (unpublished data). These results are similar to those from B7-H4VC- or B7-H4V-treated mice. Therefore, our data further support the concept that sH4 functions as a decoy molecule to block endogenous B7-H4, leading to increased autoimmune responses and exacerbated CIA.

### Neutrophils in sH4-Mediated Enhancement of Autoimmunity and CIA Exacerbation

**Figure 4:**
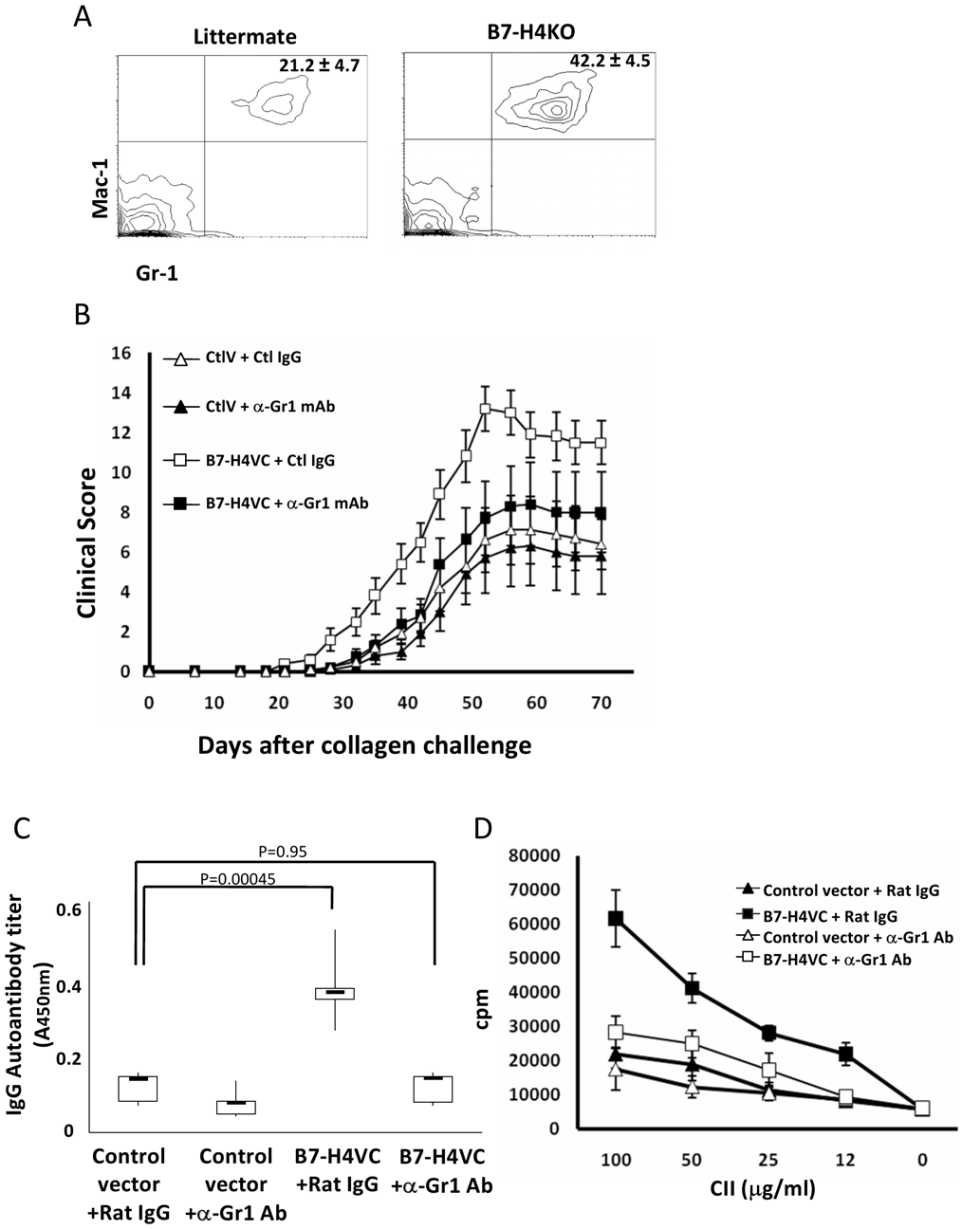
Gr-1+ cells are required for sH4-mediated exacerbation of CIA. (A) Flow cytometry analysis of peripheral blood from the B7-H4KO or littermates on Day 2 after being immunized with collagen in CFA. The percentages of Gr-1+ Mac-1+ cells are shown and the results are presented as means±standard deviation (*n* = 5). Data are representative of two independent experiments. (B) Depletion of Gr-1+ neutrophil ameliorates sH4-induced exacerbation of CIA. Mice were treated with either control (ctlV) or B7-H4VC plasmids (B7-H4VC) at day −1 and 14 of collagen immunization to induce CIA. To deplete Gr-1+ cells, mice were injected i.p. with 0.3 mg of anti-Gr-1 mAb (clone RB6-8C5) or control rat IgG every other day starting at day −1 of CIA induction and stopping at day 14. Each point represents results from a pool of ten mice and the bars are means ± 95% confidence interval. Data are representative of two independent experiments. Statistical analysis was performed by repeated ANOVA method (*p*<0.0001). The following Tukey-Kramer test showed significant difference at each point after Day 28 between the B7-H4VC+ Ctl IgG group and other three groups (*p*<0.01). (C) Depletion of Gr-1+ cells inhibited sH4-mediated production of autoantibodies to collagen. CIA mice were treated with indicated plasmids and anti-Gr-1 antibody as described in (B). Sera were sampled on day 30 after collagen immunization. Serum anti-collagen IgG level was determined by specific ELISA as described in Materials and Methods. Each group represents results from a pool of five mice. The top and bottom of each rectangular box denote the 75th and 25th percentiles, respectively, with the median shown inside the box. Error bars extending from each box represent the maximum and minimum. Data are representative of two independent experiments. Statistical analysis was performed by ANOVA method followed by the Scheffé test. (D) Depletion of Gr-1+ cells inhibited sH4-mediated proliferation of splenocytes to CII. CIA mice were treated with indicated plasmids and anti-Gr-1 antibody as described in (B). Whole splenocytes from the mice at day 30 after collagen immunization were cultured in the presence of the indicated concentrations of CII for 72 h. The cultures were pulsed with ^3^HTdR 18 h before harvest and cpm were counted by a scintillation counter. Each point represents results from three wells and data represent five independent experiments and are expressed as means ± 95% confidence interval.

Our recent study indicates that B7-H4KO mice have an accelerated response of neutrophils to infection and inflammation partially due to B7-H4-mediated growth inhibition of bone marrow–derived neutrophil progenitors [30]. These findings implicate a role of neutrophils in sH4-mediated disease exacerbation. We quantified the Gr-1+ Mac-1+ neutrophils in peripheral blood in the CIA model on day 2 after primary collagen immunization. Gr-1+ Mac-1+ cells were more frequent in B7-H4 KO mice (42.4%) than in littermate controls (21.2%) (Figure 4A). Similar results were also observed after treatment with the plasmids encoding B7-H4VC or B7-H4V (unpublished data). Because Gr-1+ cell increases precede adaptive autoimmunity, which appears at least 1 wk after exposure to CII immunization, it is thus possible that increased Gr-1+ cells may regulate subsequent development of T and B cell–mediated autoimmune response.

We next depleted Gr-1+ cells by anti-Gr-1 antibody to determine their role in CIA. Because symptoms and pathology of arthritis appear around 25–30 d after first CII immunization, CIA could be divided into induction phase (before appearance of symptoms) and effector phase (after appearance of symptoms). We first examined the effect of neutrophil depletion in sH4-mediated exacerbation of CIA. CIA mice were treated with B7-H4VC vectors and inoculated with anti-Gr-1 depleting antibody every other day from day −1 to 14. By this method, >90% of Gr-1+ cells were depleted throughout the treatment period based on flow cytometry analysis using anti-Gr1/Mac-1 mAb (unpublished data). Increased CIA clinical score induced by sH4 was eliminated by this treatment (B7-H4VC+Ctl IgG group versus B7-H4VC+anti-Gr-1 mAb group) (Figure 4B). Our result supports the hypothesis that neutrophils are required for sH4-mediated exacerbation of CIA. Furthermore, in the condition of anti-Gr-1 depletion, B7-H4VC treatment did not have a significant effect in exacerbation of CIA (Figure 4B). Interestingly, anti-Gr1 mAb did not suppress CIA induced by CII without sH4, suggesting that the neutrophil-mediated mechanism is unique for sH4. Taken together, our data indicate the sH4-mediated exacerbation of CIA is dependent on Gr-1+ cells.

We also depleted Gr-1+ cells in the effector phase of CIA to determine the role of Gr-1+ cells. In contrast to the treatment in the induction phase, depletion of Gr-1+ cells in the effector phase had only a marginal effect in B7-H4VC plasmid-induced CIA exacerbation (Figure S9). Because this treatment also inhibited CIA severity without B7-H4VC treatment (CtlV+Clt IgG versus CtlV+anti-Gr1 mAb), this marginal effect may not be sH4 dependent. In addition, even when Gr-1+ cells were depleted, B7-H4VC plasmid treatment could still induce exacerbation of CIA (CtlV+anti-Gr1 mAb versus B7-H4VC+anti-Gr1 mAb). These findings indicate that Gr-1+ cells have a minimal role in sH4-mediated exacerbation of CIA, which is in contrast to their role in the induction phase, in which depletion of Gr-1+ cells eliminated the effect of sH4 (Figure 4B).

To confirm that the depletion of Gr-1+ cells affects subsequent generation of adaptive autoimmune responses, we evaluated the anti-collagen antibody (Figure 4C) and T cell proliferation to collagen (Figure 4D) in the sH4-excerbated CIA model after depletion of Gr-1+ cells. In CIA mice treated by B7-H4VC plasmid, the depletion of Gr-1+ cells completely inhibited the production of sH4-mediated increases of autoantibodies and T cell proliferation to collagen.

### Suppression of CIA by Agonist B7-H4

While our data show that sH4 in mouse models promotes CIA, these data also support the view that endogenous B7-H4 is a checkpoint molecule in suppressing autoimmune responses. Therefore, a potential approach to suppressing autoimmune diseases is to increase the expression of B7-H4 in the form of agonist in order to engage its putative receptor. To do so, we constructed B7-H4Ig fusion protein in which the B7-H4 extracellular domain was fused to mouse IgG2a Fc portion as described previously [23],[35]. The Fc portion of B7-H4Ig could bind Fc receptor–positive cells to facilitate the agonist effect in vivo. In the control vector, signal sequence of B7-H4 was fused to murine IgG2a Fc portion. B7-H4Ig and the control vector were transfected into 293 T cells and the same level of production of Fc was confirmed with two plasmids (unpublished data).

**Figure 5:**
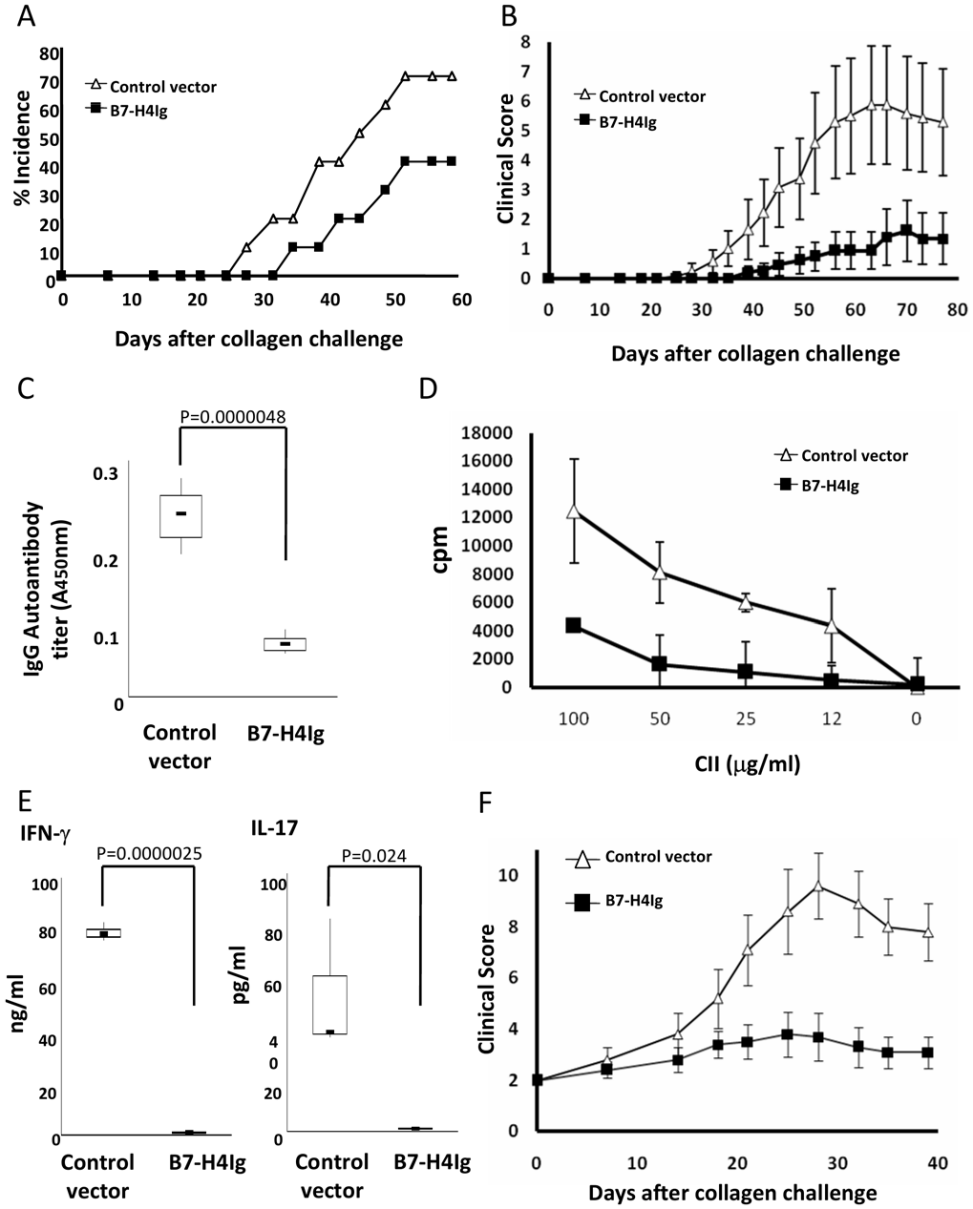
B7-H4Ig fusion protein suppresses autoimmune responses and development of CIA. (A) Inhibition of CIA incidence and (B) clinical score by treatment with the plasmid containing the gene encoding B7-H4Ig fusion protein. Mice in groups of ten were immunized with CII in CFA on day 0 and day 21 and observed daily. The incidence and clinical score were recorded based on the standard method described in Materials and Methods. Indicated plasmids were injected on day −1 and day 20 of CIA induction. Data are representative of three independent experiments and the data of clinical scores are expressed as means ± 95% confidence interval. Statistical analysis was performed by repeated ANOVA method (*p*<0.0001). The following Tukey-Kramer test showed significant difference at each points after day 33 (*p*<0.01). (C) Inhibition of autoantibodies to collagen by B7-H4Ig. CIA mice in groups of five were treated with indicated plasmids and sera were sampled at day 30 after collagen immunization. Serum anti-collagen IgG level was determined by specific sandwich ELISA as described in Materials and Methods. The top and bottom of each rectangular box denote the 75th and 25th percentiles, respectively, with the median shown inside the box. Error bars extending from each box represent the maximum and minimum. Data represent three independent experiments. The data were analyzed by unpaired Student two-tailed *t*-test. (D) Inhibition of T cell proliferation to collagen by B7-H4Ig. CIA mice in groups of five were treated with indicated plasmids as described in (A). Whole splenocytes from the mice at day 30 after collagen immunization were cultured in the presence of the indicated concentrations of CII for 72 h. The cultures were pulsed with ^3^HTdR 18 h before harvest and cpm were counted by a scintillation counter. Each point represents results from three wells. The data represent three independent experiments and are expressed as means ± 95% confidence interval. (E) Inhibition of IFN-γ and IL-17 production responding to collagen by B7-H4Ig. Spleen cells from CIA mice were stimulated as described in (D). Supernatants in the cultures at 72 h were collected and assessed for IFN-γ and IL-17 production by specific sandwich ELISA. Each group represents results from three wells. The data represent three independent experiments. The top and bottom of each rectangular box denote the 75th and 25th percentiles, respectively, with the median shown inside the box. Error bars extending from each box represent the maximum and minimum. The data were analyzed by unpaired Student two-tailed *t*-test. (F) Suppression of established CIA by treatment with the plasmid containing the gene encoding B7-H4Ig fusion protein. Mice in groups of 10 were immunized with chicken type II collagen in CFA on day 0 and day 21 and observed daily. When the mice had a clinical score 2, they were injected with indicated plasmids. The treatment was repeated once 2 wk later. The incidence and clinical score were recorded based on the standard method described in Materials and Methods. Data are representative of three independent experiments and the data of clinical scores are expressed as means ± 95% confidence interval. Statistical analysis was performed by the repeated ANOVA method (*p*<0.0001). The following Tukey-Kramer test showed significant differences at each point after day 21 (*p*<0.01).

The effect of B7-H4Ig on preventing the progression of CIA was first tested. The plasmids were injected hydrodynamically 1 d before CII immunization and the same treatment repeated 1 d before CII challenge at day 21. In comparison with the control vector, B7-H4Ig plasmid treatment significantly delayed the onset of CIA and decreased the incidence and clinical score of arthritis (Figure 5A, 5B). Furthermore, B7-H4Ig plasmid treatment suppressed the production of total IgG (Figure 5C) and IgG_1_, IgG_2a_, and IgG_2b_ autoantibodies to CII (Figure S7). Finally, proliferation of splenocytes (Figure 5D) and CD4+ T cells (Figure S8), as well as IFN-γ and IL-17 production in response to CII, were also significantly suppressed upon B7-H4Ig treatment (Figure 5E). The treatment also significantly suppressed joint swelling (Figure S4) and inhibited infiltration of inflammatory cells (Figure S5), including Gr-1+ cells and lymphocytes (unpublished data). Collectively, our results support that B7-H4Ig works as an agonist for its putative receptor to suppress both humoral and cellular autoimmunity. In addition, this method is also effective in suppressing progression of CIA.

We next evaluated whether B7-H4Ig could be applied to treat established CIA. Mice were immunized with CII on day 0 and day 21. When the mice had a clinical score of 2, which happened between 20–36 d after initial CII immunization, they were injected with B7-H4Ig or control plasmids and the same treatments were repeated once 2 wk later. As shown in Figure 5F, B7-H4Ig plasmid treatment significantly suppressed progression of CIA. Although this treatment did not cure the mice, severity of CIA was significantly suppressed up to 40 d after initial treatment.

## Discussion

sH4 is found in sera of approximately two-thirds of the RA patients we sampled, and the concentration of sH4 appears to correlate with activity of disease. In the CIA mouse model, we found evidence that sH4 acts as a decoy molecule to block suppressive functions of endogenous B7-H4, leading to enhanced autoimmune responses and exacerbation of CIA. We also demonstrated that agonist B7-H4Ig is a potent inhibitor of CIA progression. Therefore, our findings may have implications for prediction of RA disease progression and potential novel therapeutic approaches to RA.

sH4 is approximately 50 kDa by Western blot analysis (Figure 1B), which is larger than the extracellular domain of monomeric B7-H4 molecule in denatured conditions; the predicted size of B7-H4 is 26 kDa. This size discrepancy could be due to glycosylation, since B7-H4 has several potential glycosylation sites. This is consistent with a recent report showing that sH4 is about 50 kDa [28]. The mechanism for the generation of sH4, however, remains unknown. One possibility is enzymatic cleavage of the entire extracellular portion of B7-H4. In our preliminary study, 293 T cells transfected with full-length B7-H4 cDNA release sH4 into culture supernatants, and this secretion could be inhibited by incubation with various proteases inhibitors (Azuma et al., unpublished data). Our results also indicate sH4 containing only the IgV portion is sufficient to exacerbate autoimmune diseases, because B7-H4V has similar effects to B7-H4VC. This is consistent with our previous findings that the binding site for its putative receptor is located in the IgV domain based on a computer-generated model [23]. B7-H4 was suggested to be a glycosylphosphatidylinositol (GPI)-anchoring protein [26], which could become soluble by detaching its anchoring [36]. However, a recent study shows that B7-H4 is a transmembrane protein [37]. Another possibility is that sH4 is made from an alternatively spliced form. Several molecules in the immunoglobulin superfamily have been shown to display soluble forms [22],[38]–[40] and these soluble molecules, including CD80, CD86, and PD-1, are made by splicing variants [22],[38],[39]. Therefore, this possibility could not be excluded at the present time.

We found elevated sH4 in 65% of RA patients in our study population. These figures, however, might represent an underestimation of the true proportions of patients with sH4 elevations. Each patient was sampled only once, at their clinic visit, and the concentration of serum sH4 may vary during disease progression. In addition, all patients were already under treatment with various regimens (Table S1) and such treatment may affect the production of sH4. A kinetic study with multiple blood draws in newly diagnosed patients in a prospective setting will be ideal to address these issues. Nevertheless, our data show that patients with highly active RA have significantly higher levels of sH4 than the patients in remission or with low disease activity. It appears to be a trend that a higher level of sH4 is associated with increased RA activity. However, sH4 in the patients with moderate disease activity was not significantly different from the patients in remission (Figure 1C). This observation may need to be further tested by increasing the number of patients in each study group to increase statistical power. While our data suggest a correlation of elevated sH4 in sera to disease activity and severity, it is difficult to determine the role of sH4 in the pathogenesis of human RA. To help address this issue, we applied mouse CIA models, a well characterized mouse model for human RA, to further address if sH4 could promote disease activity. Using a hydrodynamic injection method to express B7-H4 plasmid in vivo, we demonstrated that expression of sH4 promotes autoimmune responses, leading to progression of CIA, and similar results are also observed in B7-H4–deficient mice. In contrast, introduction of the plasmid encoding B7-H4Ig could prevent disease induction of CIA. More strikingly, treatment of ongoing autoimmune disease by B7-H4Ig was also effective. While our results in the mouse CIA model suggest a role of B7-H4 in pathogenesis of CIA, one should be cautious because the CIA model does not mimic human RA in all aspects, and it remains to be tested whether sH4 participates in the development of human RA. Elevation of sH4 may also serve as a disease severity marker because a significant fraction of RA patients who have elevated sH4 had more severe RA than those without sH4 in their sera. To establish this as a biomarker, however, would require further studies including more patients and preferably at their primary visit.

The effect of endogenous B7-H4 in the inhibition of T and B cell–mediated autoimmune responses in the CIA mouse model is evident as shown in increased T and B cell responses by B7-H4 blocking. This is shown in vivo by transgenic expression of sH4 or B7-H4 genetic knockout. In addition, expression of agonist B7-H4Ig fusion protein significantly inhibits T cell and autoantibody-mediated responses. Consistent with previously published observations from different laboratories, our observation thus supports a critical role of B7-H4 in the inhibition of adaptive immunity, leading to suppression of autoimmune diseases in the CIA model. It has been shown that B7-H4 executes its effect by inhibiting cell cycle progression of T cells in the presence of antigen stimulation [23] while a direct effect of B7-H4 on B cells has not been documented. In addition to T and B cell immunity, our results also implicate a role of Gr-1+ cells in sH4-induced exacerbation of CIA. First, neutrophil numbers increase rapidly in peripheral blood after sH4 exposure in CIA (Figure 4A and Azuma et al., unpublished observation). Second, infiltration of Gr-1+ cells increases in various disease organs (Figure S5 and Azuma et al., unpublished observation). Finally, depletion of Gr-1+ cells eliminates the effect of sH4. Our experiments indicate that Gr-1+ cells contribute to the induction phase of sH4-mediated enhancement of autoimmunity and disease exacerbation while its role in the effector phase is minimal (Figures 4B and S9). While Gr-1+ cells are largely neutrophils, they may also contain a small number of plasmatoid dendritic cells [41]. It is tempting to speculate that secretion of sH4 in RA first enhances innate immunity by stimulation of Gr-1+ cells and subsequently facilitates adaptive autoimmunity. It is unclear at the present time how Gr-1+ cells enhance autoimmune immune responses, but various studies indicate that Gr-1+ cells may do so by inducing maturation of dendritic cells [42]. Because sH4 could be present in these patients for a long period of time, our findings suggest a possible mechanism for persistent inflammation. In addition to their antimicrobial effect, Gr-1+ neutrophils have been implicated in autoimmune disease and chronic inflammation [4],[5],[6]. In this context, neutrophil infiltration is considered to play an important role in arthritis pathology in a number of animal models including K/BxN serum-induced arthritis, anti-type II collagen antibody induced arthritis, Lyme arthritis, adjuvant arthritis, streptococcal cell wall–induced arthritis, and CIA [9]–[14],[43],[44]. In these models, neutrophil depletion or neutrophil-derived mediators could ameliorate the severity of arthritis. Neutrophils can synthesize a variety of cytokines and chemokines [16],[17]. Chemokines released by Gr-1+ cells could also amplify inflammation by recruiting more Gr-1+ cells and other inflammatory cells into the inflammatory site [45]–[47]. Although our results suggest a potential role of Gr-1+ cells on the induction of autoimmunity, depletion of Gr-1+ cells does not eliminate the induction of CIA by antigen+adjuvant (Figure 4B). Therefore, a neutrophil-dependent mechanism may operate only in the presence of sH4.

Increased neutrophils in peripheral blood have been found in RA patients and this elevation was shown to be associated with more severe disease [48], suggesting a potential role of neutrophils in the exacerbation of RA. Neutrophils are mostly found in RA patients' synovial fluid of the involved joints, which is different from the CIA model in which infiltration of neutrophils in the joint lesion is evident. While it is impossible at the present time to establish a causal relationship among increased sH4/neutrophils and severe RA diseases clinically, our findings suggest this possibility and encourage further study to elucidate this issue.

In summary, our findings in the CIA mouse model support a critical role for B7-H4 as a checkpoint molecule and provide a possible explanation for the pathogenesis and progression in a significant fraction of patients with RA. Production of decoy B7-H4 in these patients may facilitate disease progression by enhancing innate immune responses, leading to amplified adaptive autoimmune responses against self antigens. In this regard, sH4 might be useful as a biomarker for diagnosis and monitoring of the progression of RA. On the other hand, sH4 may be potentially utilized to enhance anti-tumor or anti-viral immunities. Our experiments also implicate agonist forms of B7-H4 such as B7-H4Ig as a new venue for the potential treatment of autoimmune diseases.

## Supporting Information

Figure S1 Association of sH4 and the levels of CRP in patients diagnosed with RA. The data are a summary of 68 RA patients and are analyzed by Spearman's rank test. *y* = 36.9*x*−95.9, *R* ^2^ = 0.473, *p*<0.001.
(0.51 MB TIF)

Figure S2 The levels of sH4 in CIA mice. Mice were immunized with chicken type II collagen in CFA on day 0 and day 21 and sera were collected from the mice at the indicated time points and subjected to ELISA analysis as described in Materials and Methods. Concentration of serum sH4 was determined by comparison with purified B7-H4Ig protein. Each point represents results from a pool of five mice. Data represent two independent experiments and are expressed as means ± 95% confidence interval.
(0.51 MB TIF)

Figure S3 Kinetics of sH4 in mouse sera after hydrodynamic injection of the plasmids. Sera were collected from the mice at the indicated time points and subjected to ELISA analysis as described in Materials and Methods. Concentration of serum sH4 was determined by comparison with purified B7-H4Ig protein. Each point represents results from a pool of five mice. Data represent two independent experiments and are expressed as means ± 95% confidence interval.
(0.54 MB TIF)

Figure S4 Gross appearance of paws of mice after collagen immunization. Joint swelling of the normal (left) or CIA mice treated with control vector, B7-H4VC, or B7-H4Ig plasmids on day 45.
(3.11 MB TIF)

Figure S5 Histology of paws of mice after collagen immunization. Hind paws from the normal (left) or CIA mice were treated with control vector, B7-H4VC, or B7-H4Ig plasmids. On day 35, tissue sections from the metatarsophalangeal joints of the mice were prepared and processed by hematoxylin and eosin staining (magnification, ×100).
(2.94 MB TIF)

Figure S6 Clinical score of mice with CIA treated by control vector. Mice were immunized with CII in CFA on day 0 and day 21. The clinical scores were recorded based on the standards described in Methods. Mice in groups of ten were injected with the indicated plasmids on day −1 and day 20. Data are representative of two independent experiments and are expressed as means ± 95% confidence interval. Statistical analysis was performed by the repeated ANOVA method (*p*<0.0001). The following Tukey-Kramer test showed significant differences at each points after day 28 between the Flag group and other two groups (*p*<0.01).
(0.57 MB TIF)

Figure S7 Serum titers of autoantibodies. Serum levels of anti-CII IgG1, IgG2a, and IgG2b from the groups of CIA mice (five per group) treated with control vector, B7-H4V, B7-H4VC, or B7-H4Ig were measured by specific sandwich ELISA on day 30 and expressed as means ± 95% confidence interval. Statistical analysis was performed by the ANOVA method followed by the Scheffé test.
(0.68 MB TIF)

Figure S8 The proliferation of CD4+ T cells against collagen type II from CIA mice after exposure to sH4. CD4+T cells were purified from spleens 30 d after CII immunization and stimulated in vitro by indicated concentration of CII for 72 h before harvesting. CIA mice pre-treated with control vector, B7-H4V, B7-H4VC, or B7-H4Ig prior analysis. The data are presented as means ± 95% confidence interval.
(0.54 MB TIF)

Figure S9 Effect of neutrophil depletion in the effector phase in sH4-mediated exacerbation of CIA. CIA mice were treated with the indicated plasmids at day −1 and day 14. To deplete Gr-1+ cells, mice in groups of ten were injected i.p. with 0.3 mg of anti-Gr-1 mAb (clone RB6-8C5) or control rat IgG every other day from day 20, and this treatment was stopped at day 35 after collagen immunization. The bars are means ± 95% confidence interval. Data are representative of two independent experiments. Statistical analysis was performed by repeated ANOVA method (*p*<0.0001). The following Tukey-Kramer test showed significant difference at each points after day 28 between the B7-H4VC+anti-Gr1 mAb group and CtlV+anti-Gr1 mAb group (*p*<0.01).
(0.58 MB TIF)

Table S1 Characteristics of patients diagnosed with rheumatoid arthritis.
(0.84 MB TIF)

## Abbreviations

ANA: anti-nuclear antibody
ANC: absolute neutrophil count
B7-H4: B7 homolog 4
B7-H4Ig: B7-H4-immunoglobulin Fc fusion protein
CIA: collagen-induced arthritis
CII: type II chicken collagen
CRP: C-reactive protein
DAS: disease activity score
dsDNA: double-stranded DNA
G-CSF: granulocyte colony stimulating factor
HD: healthy donor
HRP: horseradish peroxidase
IL: interleukin
KO: knockout
mAb: monoclonal antibody
PD-1: programmed death one
RA: rheumatoid arthritis
sH4: soluble B7H4
TNF-α: tumor necrosis factor-alpha
