# Supplementary figures and images for: Potential Role of Decoy B7-H4 in the Pathogenesis of Rheumatoid Arthritis: A Mouse Model Informed by Clinical Data
Source: PLoS Med. 2009 Oct 20;6(10):e1000166. doi: 10.1371/journal.pmed.1000166 (PMC2760136; doi:10.1371/journal.pmed.1000166)

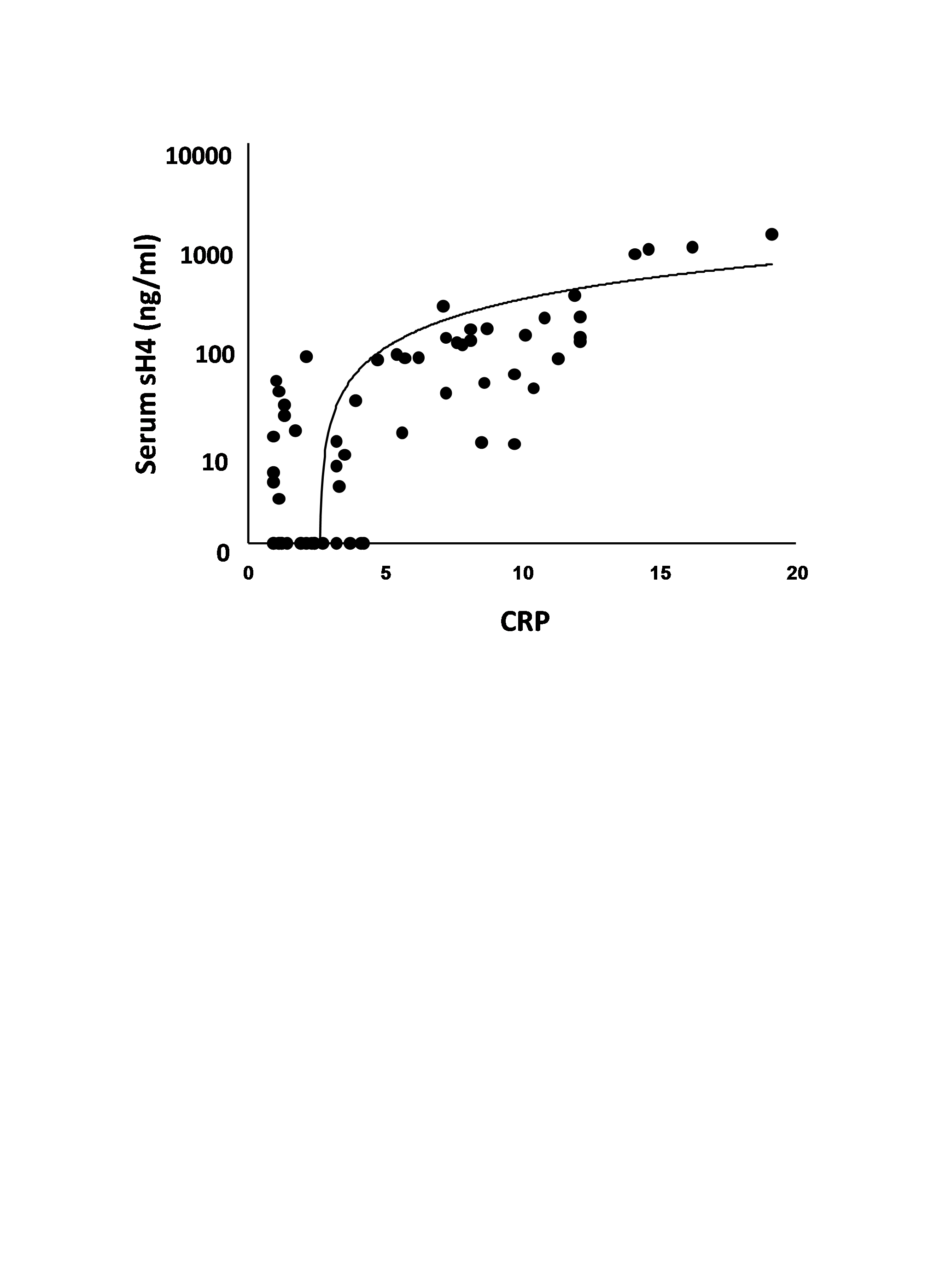

Supplement: Figure S1 — Association of sH4 and the levels of CRP in patients diagnosed with RA. The data are a summary of 68 RA patients and are analyzed by Spearman's rank test. y = 36.9x−95.9, R 2 = 0.473, p<0.001. (0.51 MB TIF) [file pmed.1000166.s001.tif]

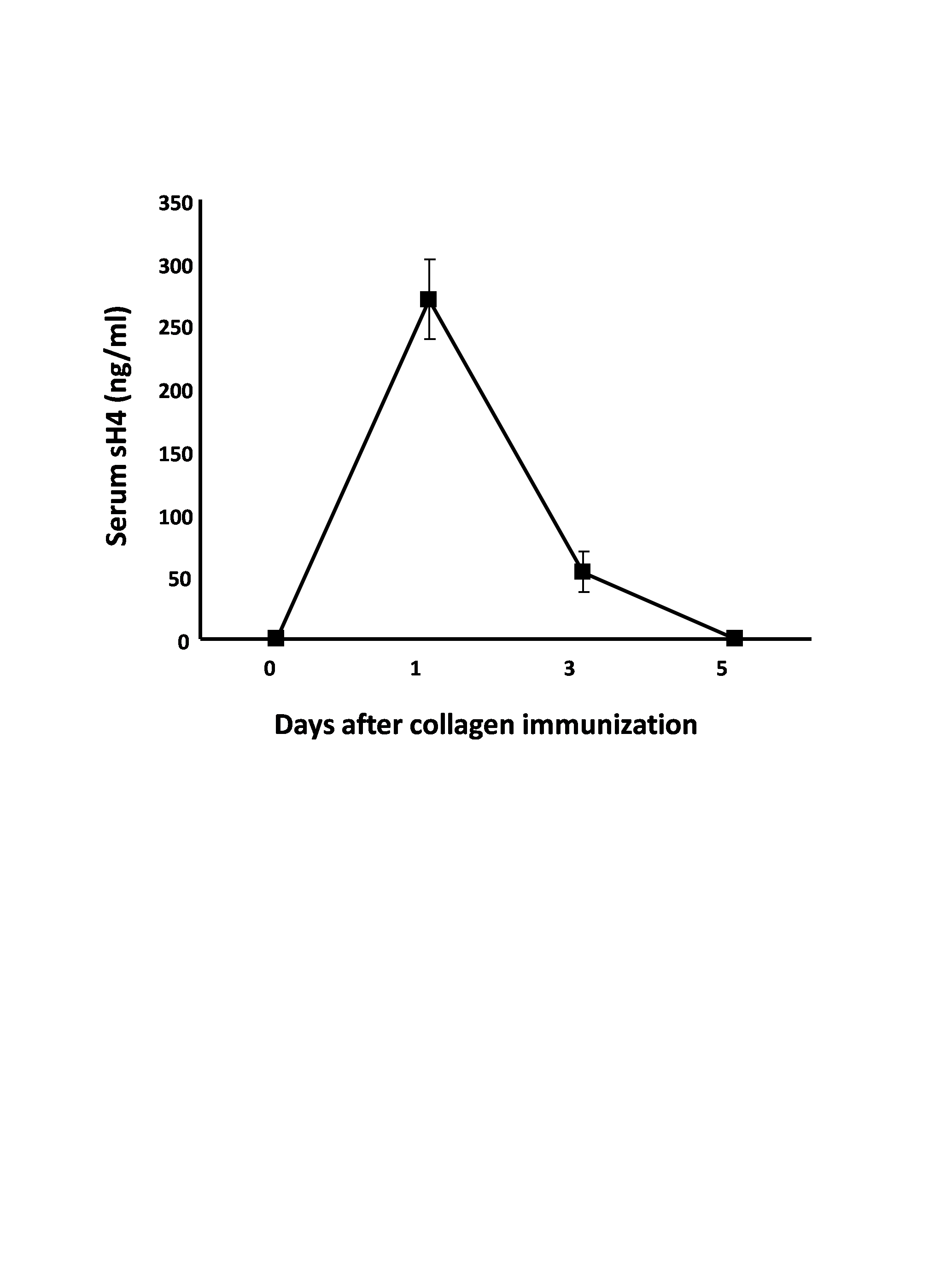

Supplement: Figure S2 — The levels of sH4 in CIA mice. Mice were immunized with chicken type II collagen in CFA on day 0 and day 21 and sera were collected from the mice at the indicated time points and subjected to ELISA analysis as described in Materials and Methods. Concentration of serum sH4 was determined by comparison with purified B7-H4Ig protein. Each point represents results from a pool of five mice. Data represent two independent experiments and are expressed as means ± 95% confidence interval. (0.51 MB TIF) [file pmed.1000166.s002.tif]

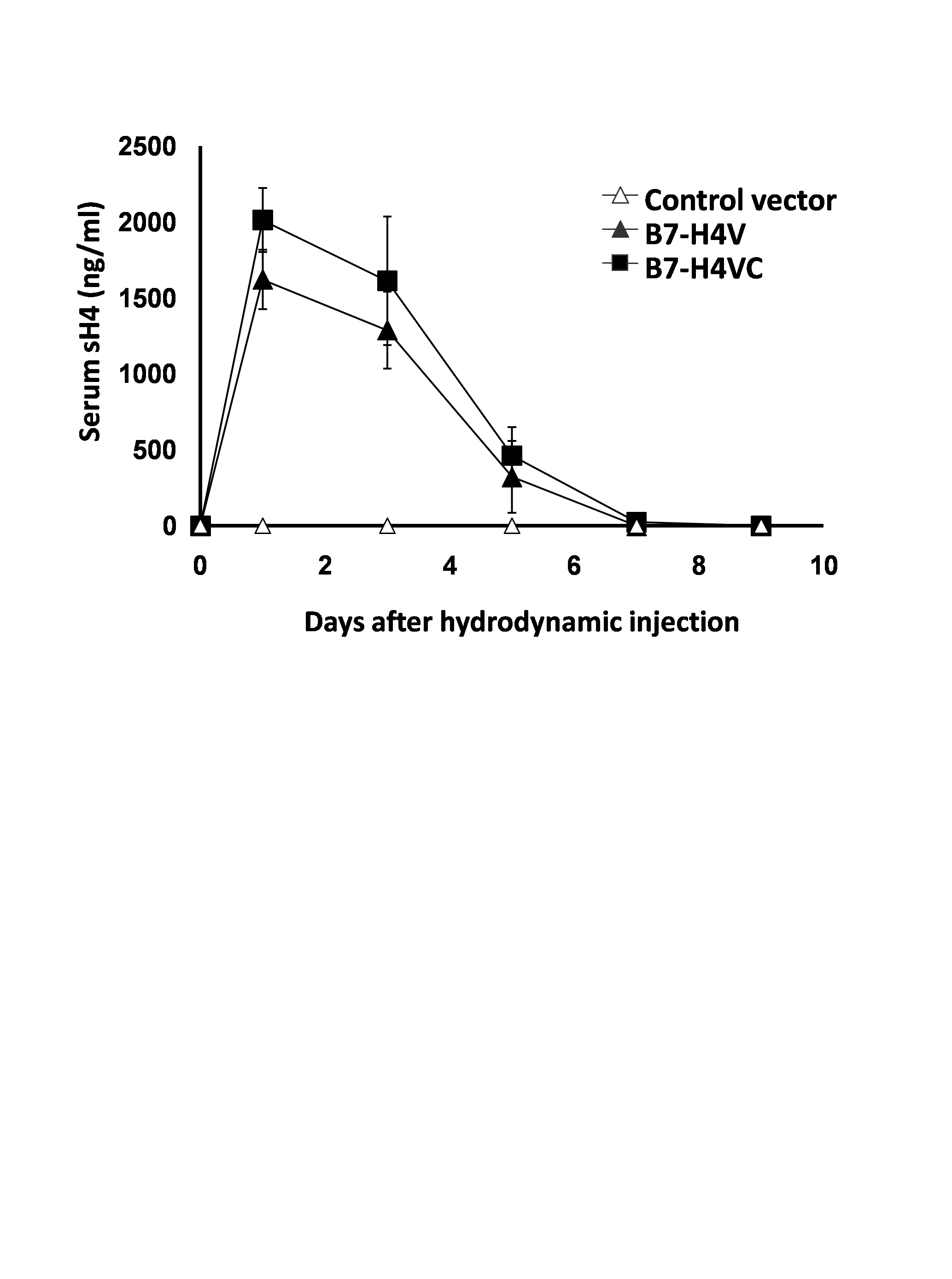

Supplement: Figure S3 — Kinetics of sH4 in mouse sera after hydrodynamic injection of the plasmids. Sera were collected from the mice at the indicated time points and subjected to ELISA analysis as described in Materials and Methods. Concentration of serum sH4 was determined by comparison with purified B7-H4Ig protein. Each point represents results from a pool of five mice. Data represent two independent experiments and are expressed as means ± 95% confidence interval. (0.54 MB TIF) [file pmed.1000166.s003.tif]

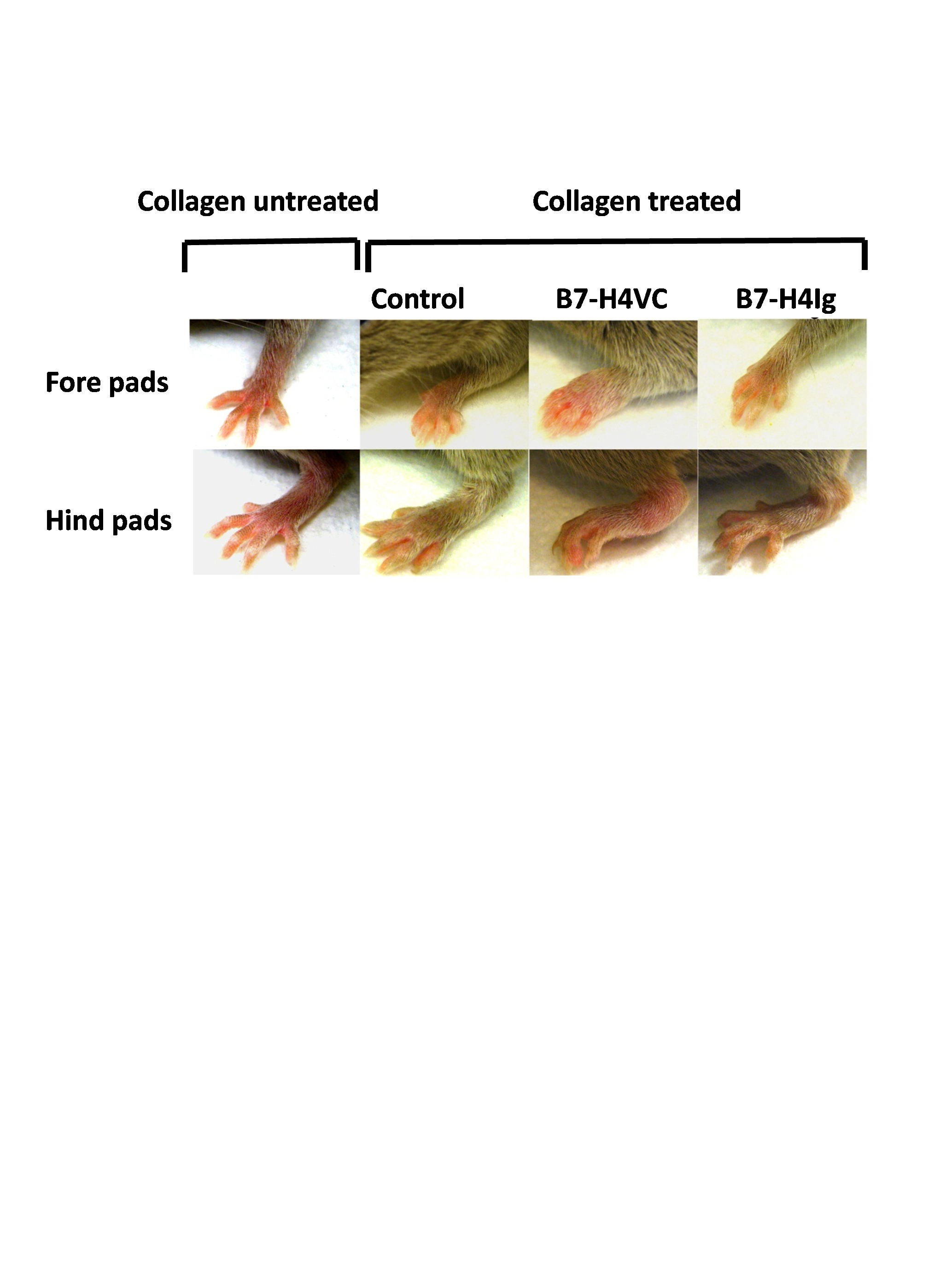

Supplement: Figure S4 — Gross appearance of paws of mice after collagen immunization. Joint swelling of the normal (left) or CIA mice treated with control vector, B7-H4VC, or B7-H4Ig plasmids on day 45. (3.11 MB TIF) [file pmed.1000166.s004.tif]

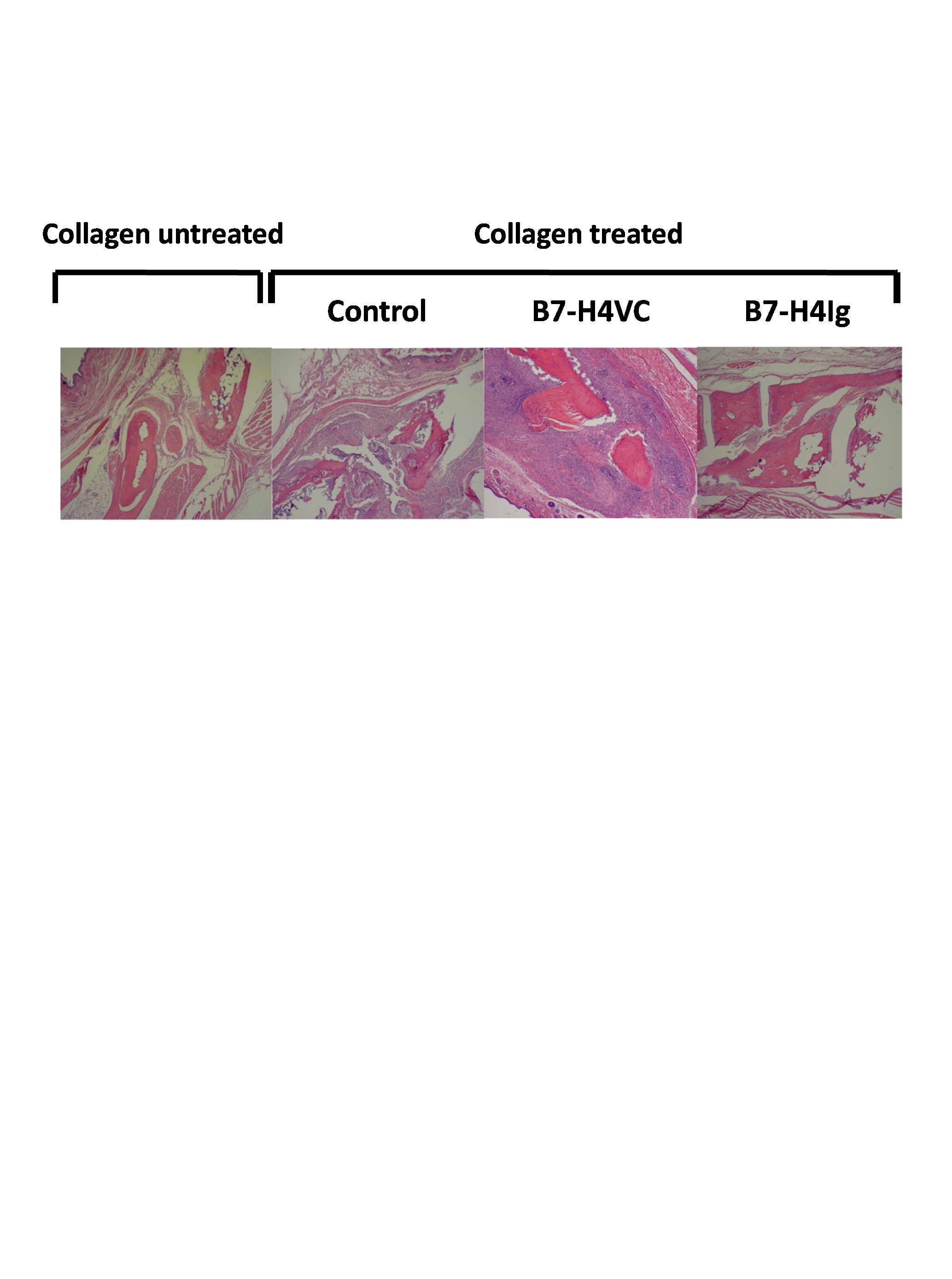

Supplement: Figure S5 — Histology of paws of mice after collagen immunization. Hind paws from the normal (left) or CIA mice were treated with control vector, B7-H4VC, or B7-H4Ig plasmids. On day 35, tissue sections from the metatarsophalangeal joints of the mice were prepared and processed by hematoxylin and eosin staining (magnification, ×100). (2.94 MB TIF) [file pmed.1000166.s005.tif]

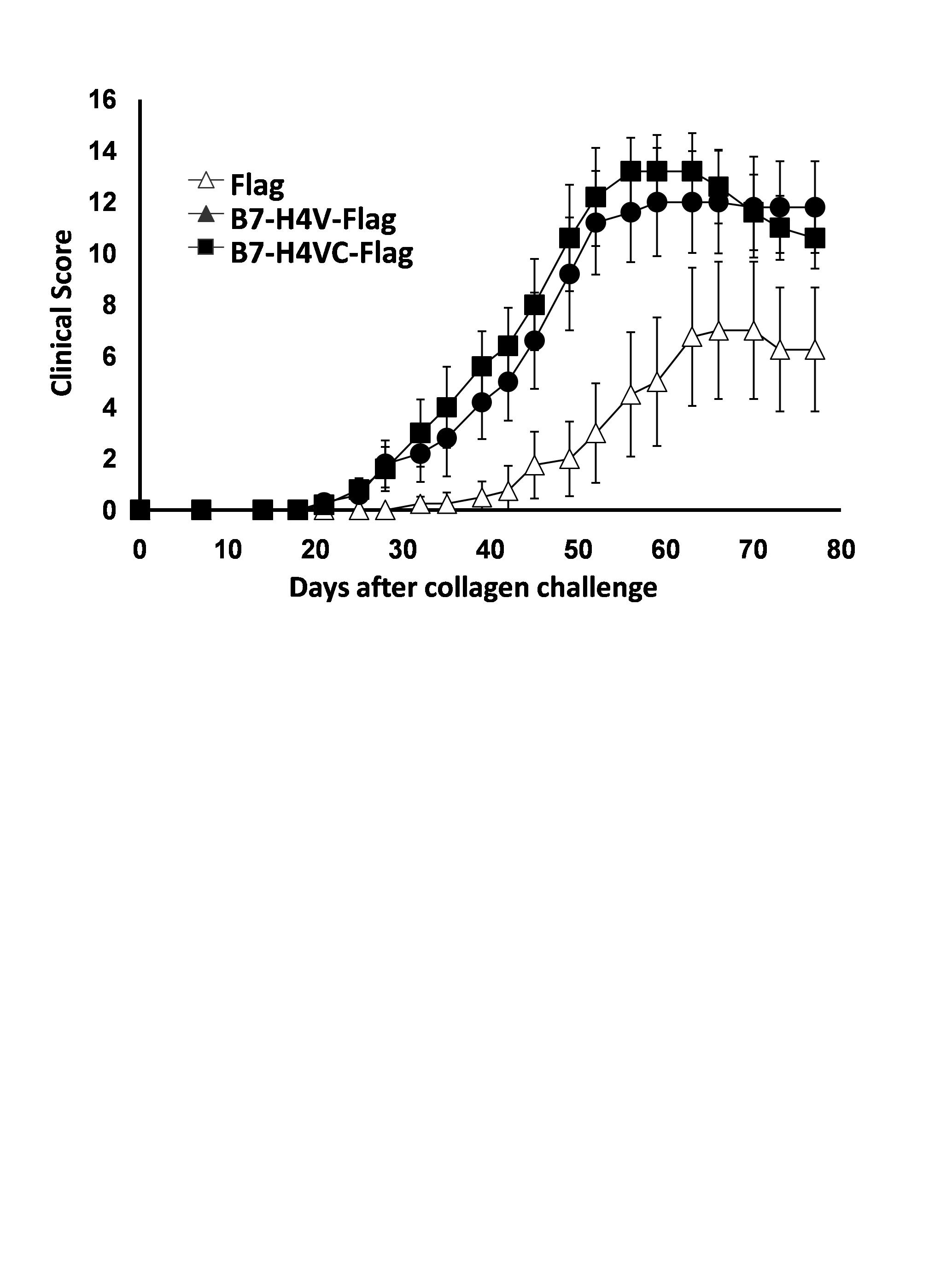

Supplement: Figure S6 — Clinical score of mice with CIA treated by control vector. Mice were immunized with CII in CFA on day 0 and day 21. The clinical scores were recorded based on the standards described in Methods. Mice in groups of ten were injected with the indicated plasmids on day −1 and day 20. Data are representative of two independent experiments and are expressed as means ± 95% confidence interval. Statistical analysis was performed by the repeated ANOVA method (p<0.0001). The following Tukey-Kramer test showed significant differences at each points after day 28 between the Flag group and other two groups (p<0.01). (0.57 MB TIF) [file pmed.1000166.s006.tif]

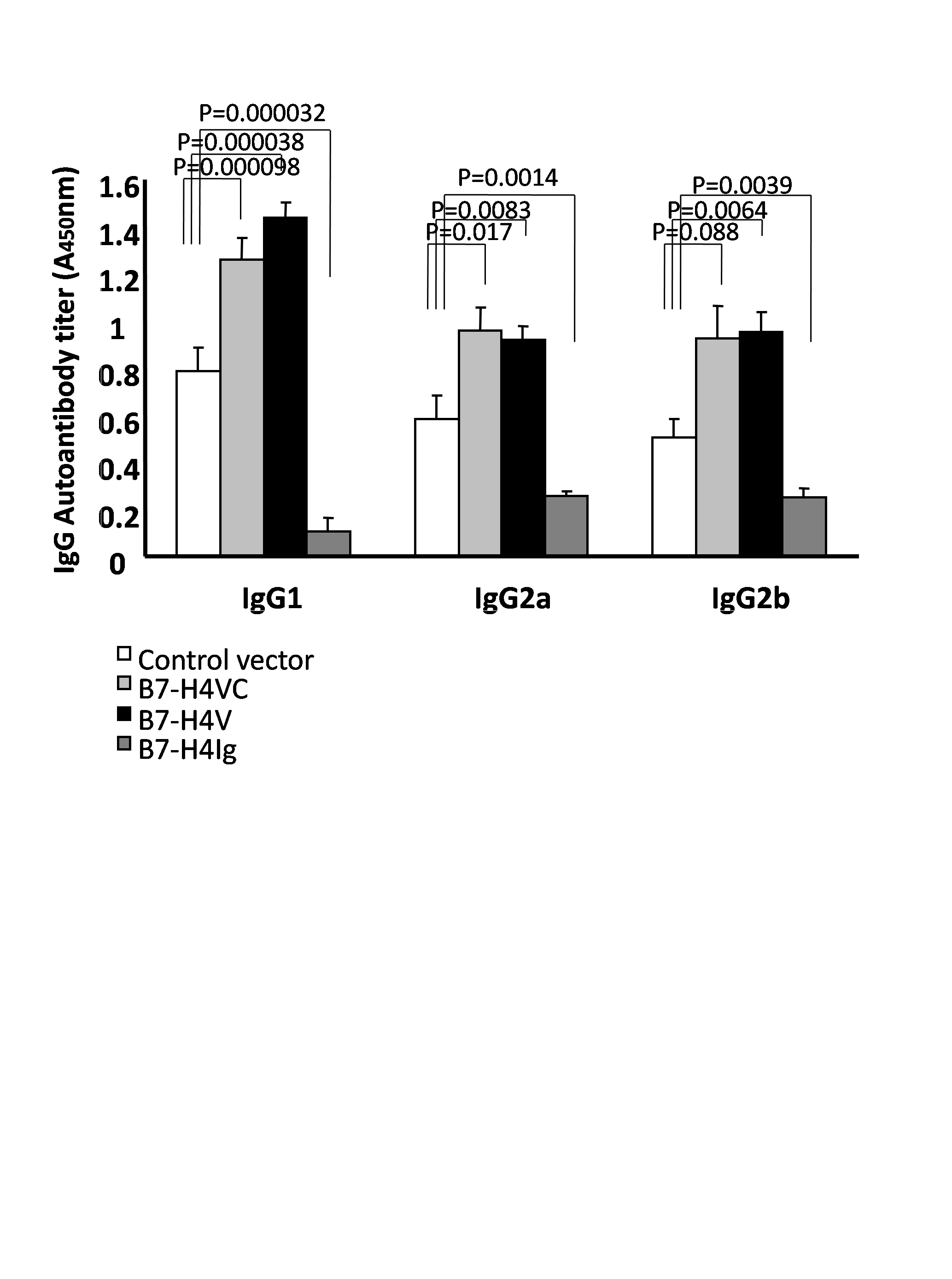

Supplement: Figure S7 — Serum titers of autoantibodies. Serum levels of anti-CII IgG1, IgG2a, and IgG2b from the groups of CIA mice (five per group) treated with control vector, B7-H4V, B7-H4VC, or B7-H4Ig were measured by specific sandwich ELISA on day 30 and expressed as means ± 95% confidence interval. Statistical analysis was performed by the ANOVA method followed by the Scheffé test. (0.68 MB TIF) [file pmed.1000166.s007.tif]

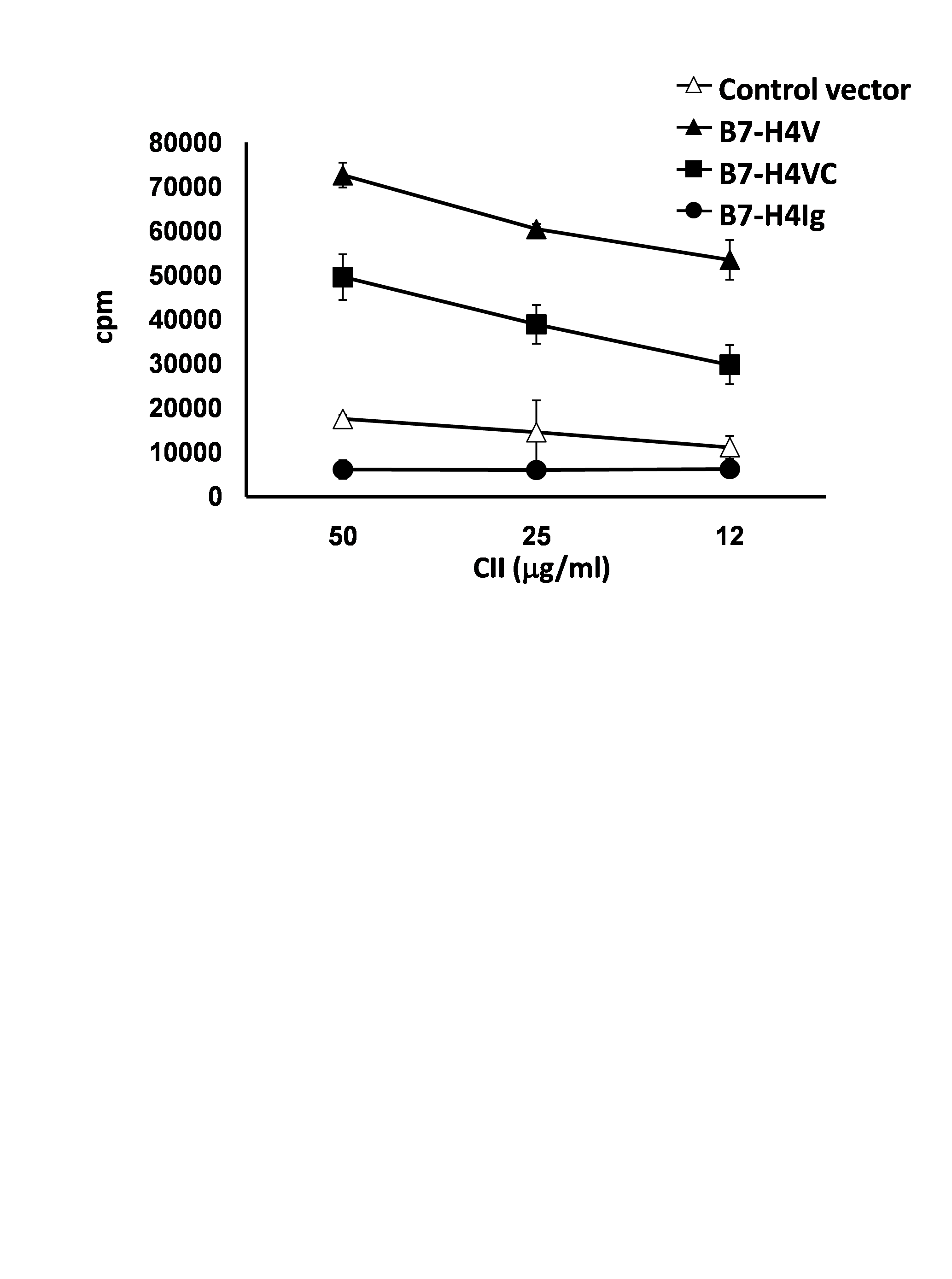

Supplement: Figure S8 — The proliferation of CD4+ T cells against collagen type II from CIA mice after exposure to sH4. CD4+T cells were purified from spleens 30 d after CII immunization and stimulated in vitro by indicated concentration of CII for 72 h before harvesting. CIA mice pre-treated with control vector, B7-H4V, B7-H4VC, or B7-H4Ig prior analysis. The data are presented as means ± 95% confidence interval. (0.54 MB TIF) [file pmed.1000166.s008.tif]

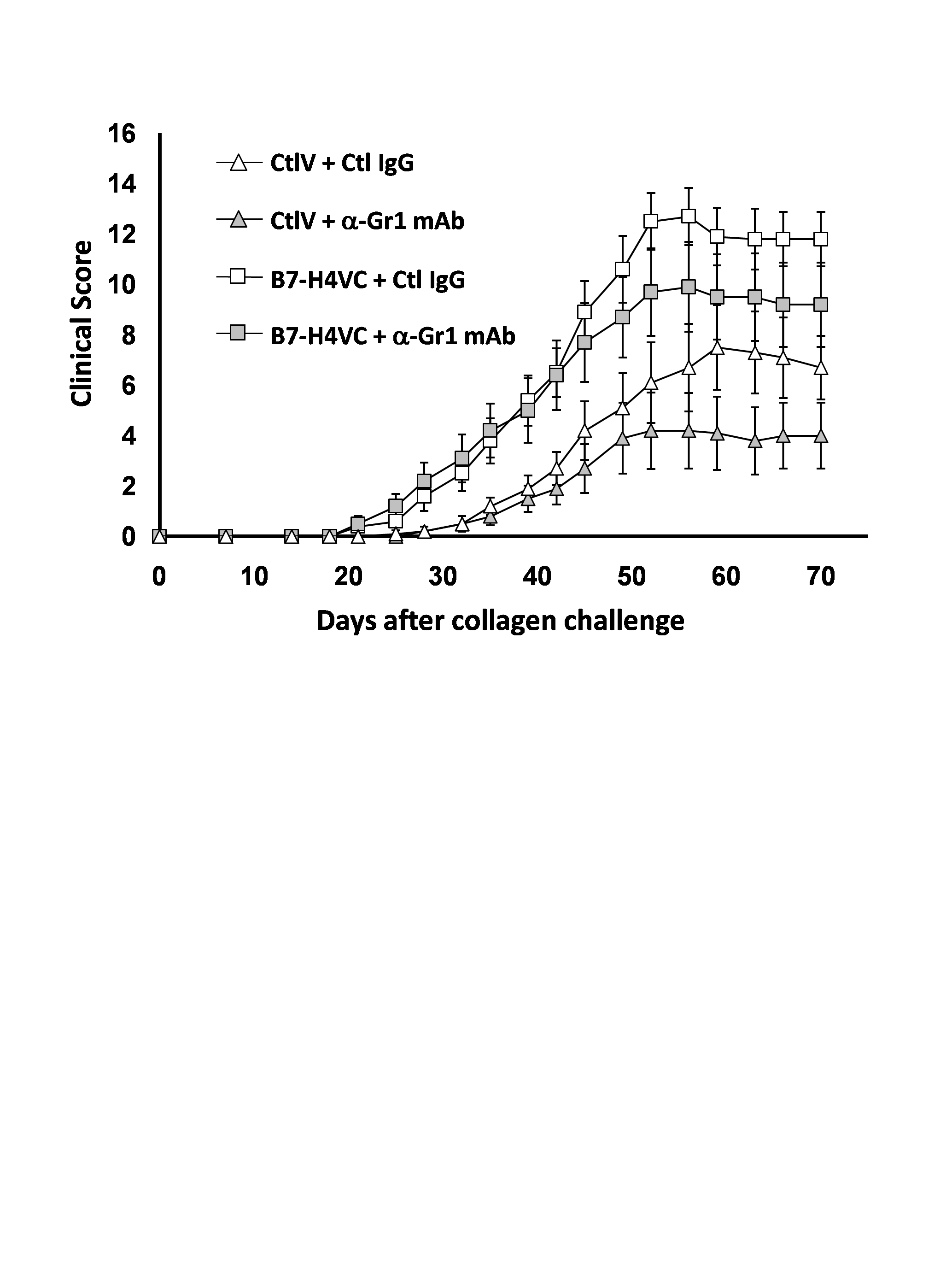

Supplement: Figure S9 — Effect of neutrophil depletion in the effector phase in sH4-mediated exacerbation of CIA. CIA mice were treated with the indicated plasmids at day −1 and day 14. To deplete Gr-1+ cells, mice in groups of ten were injected i.p. with 0.3 mg of anti-Gr-1 mAb (clone RB6-8C5) or control rat IgG every other day from day 20, and this treatment was stopped at day 35 after collagen immunization. The bars are means ± 95% confidence interval. Data are representative of two independent experiments. Statistical analysis was performed by repeated ANOVA method (p<0.0001). The following Tukey-Kramer test showed significant difference at each points after day 28 between the B7-H4VC+anti-Gr1 mAb group and CtlV+anti-Gr1 mAb group (p<0.01). (0.58 MB TIF) [file pmed.1000166.s009.tif]

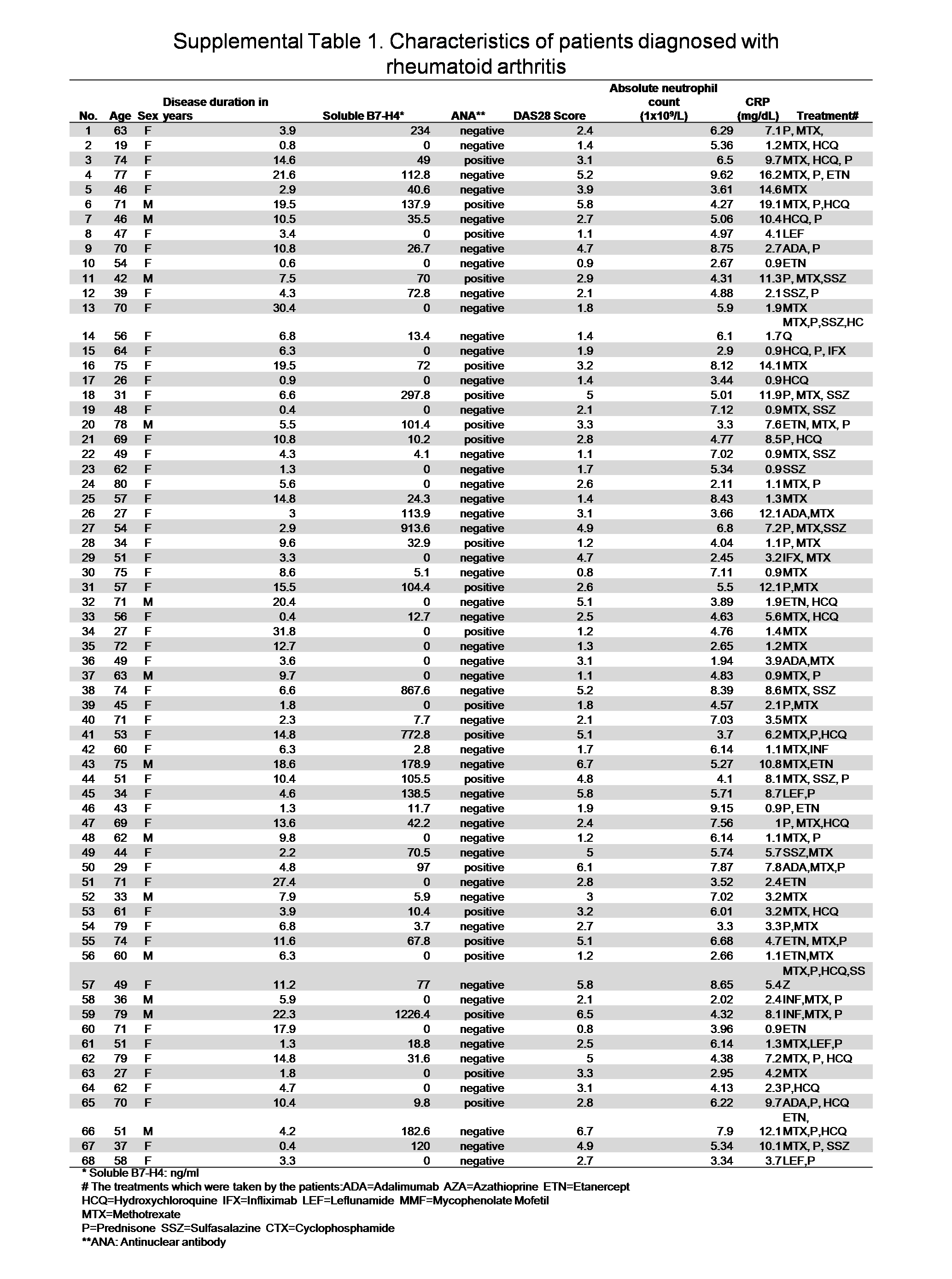

Supplement: Table S1 — Characteristics of patients diagnosed with rheumatoid arthritis. (0.84 MB TIF) [file pmed.1000166.s010.tif]
